# Coastlines retreat tipping point under storm climate changes

**DOI:** 10.1038/s41598-026-40886-9

**Published:** 2026-02-24

**Authors:** Marius Aparicio, Rafael Almar, Laurent Lacaze, Nicolas Robin

**Affiliations:** 1 Univ Toulouse, Toulouse INP, CNRS, IMFT, Toulouse, France; 2 LEGOS (CNRS-IRD-CNES-University of Toulouse), Toulouse, France; 3 CEFREM Université de Perpignan Via Domitia, 52 Avenue Paul Alduy, Perpignan, 66000 France

**Keywords:** Climate sciences, Environmental sciences, Natural hazards, Ocean sciences

## Abstract

Projected changes in ocean–atmosphere coupling under global warming suggest an intensification of storm climates, which, combined with sea-level rise, poses profound challenges to the resilience of sandy shorelines. Therefore, the definition of relevant indicators assessing beach response regimes to wave climate is crucial for future forecasts Here, we analyze 23 years of satellite-derived shoreline positions together with offshore wave data to quantify storm-induced erosion and post-storm recovery tendencies at synoptic scales. Our approach integrates statistically robust storm composites, compared against *in situ* observations from six sites worldwide, and demonstrates that daily storm-induced shoreline dynamics can be inferred from monthly global shoreline datasets. By extending the analysis using 60-year of wave reanalysis, we identify a critical threshold beyond which shoreline evolution shifts from a seasonal to a storm-dominated regime, leading to persistent erosion trajectories. Since the late 1950s, the proportion of storm-dominated beaches has increased by $\sim$2% globally, with pronounced hot-spots emerging. While local beach morphology remain essential to fully resolve coastal dynamics, our findings reveal coherent large-scale tendencies that complement site-specific surveys and provide a global framework to guide targeted field efforts. These results highlight the pivotal role of storm regime shifts in shaping the future evolution of sandy shorelines.

## Introduction

Coastal storms are among the most dynamic and disruptive drivers of shoreline change, with cascading effects on ecosystems, infrastructure, local economies, and beach management^1–3^. Anticipating their occurrence remains challenging^4,5^, as does quantifying their geomorphic impact, given the complex interplay between storm characteristics (e.g., duration, sequencing), pre-existing beach morphology, and post-storm recovery mechanisms$^{?,}$^6–8^. Under climate change, storms are projected to intensify as atmospheric energy increases, while sea-level rise continues to accelerate shoreline retreat^9–12^. Together, these forcings are expected to alter sediment transport regimes and amplify beach erosion worldwide^13–17^. As extreme events exert greater influence on sediment redistribution, understanding not only erosion but also the capacity for post-storm recovery has become critical for anticipating shoreline trajectories and guiding adaptive strategies.

The inherently non-linear response of beaches makes generalization difficult^18^. Most previous studies have focused on individual events^19^ or storm clusters^20,21^, with a stronger emphasis on erosion than recovery. This imbalance reflects the practical difficulty of maintaining high-frequency monitoring across the full storm cycle. Remote sensing now offers new opportunities to address this challenge, by providing consistent observations of storm impacts over extended spatial and temporal domains.

Recent developments in satellite-derived shoreline (SDS) datasets have enabled unprecedented exploration of beach dynamics at regional to global scales^22–31^. Despite their coarse temporal resolution (typically monthly) and known uncertainties, these datasets have proven valuable for detecting large-scale morphodynamic patterns that were previously inaccessible. Their broad spatial coverage and availability make them an efficient tool to identify regional tendencies, especially when used in complement to detailed site-based monitoring^32–35^. Nonetheless, the extent to which coarse-scale SDS analyses can be linked to local processes remains debated^11,12,22,36,37^. Here, we address the paucity of data on the resilience of microtidal sandy beaches to storms by combining 23 years (1993–2016) of SDS positions with six decades (1958–2018) of offshore wave climate. Using statistically resolved storm composites, benchmarked against six *in situ* sites across multiple basins, we identify consistent large-scale patterns of storm-induced erosion and post-storm recovery. From these patterns emerges a potential tipping point in storm frequency, beyond which shoreline response shifts from seasonal wave control to a storm-dominated regime. We stress that this threshold should not be interpreted as a deterministic predictor at individual sites, but rather as a first-order statistical tendency detectable in coarse datasets. Framed in this way, the results provide a complementary perspective to local studies, highlight potential regional hotspots, and offer a hypothesis-generating framework for testing shoreline resilience under accelerating climate pressures.

## Results

### Coastal sensitivity to storms

The skewness (*S*) and kurtosis (*K*) of offshore significant wave height ($H_s$) distributions highlight a powerful lens through which to assess the disequilibrium between mean and storm conditions along the world’s coastlines, revealing critical spatial patterns in storm wave that are not captured by conventional frequency-based indicators (see Supplementary Material, Fig. SM13). Regions with low skewness (0 $< S<$ 1) correspond to areas experiencing the lowest storm frequencies per year, primarily within the intertropical band. In contrast, positively skewed distributions ($S>$ 1) dominate latitudes with high storm frequencies. However, some high-energy coastlines within major storm tracks, such as the west coasts of North American (NAWC) and Europe (WE), display surprisingly moderate skewness. In these settings, the consistency and intensity of wave activity result in relatively homogeneous wave conditions, diminishing statistical contrasts between mean and extreme events. Conversely, semi-enclosed seas like the Caribbean, the Mediterranean and the East sea where ambient wave energy is typically low-to-moderate, exhibit high skewness. This indicates that storm events may be morphodynamically significant, standing in stark contrast to background conditions. Kurtosis patterns closely mirror those of skewness, with elevated values pointing toward regions where storm energy deviates sharply from statistical norms, suggesting a dominance of extreme events in shaping shoreline trajectories. In contrast, regions like the NWA and WE, while subject to frequent storms^38^, exhibit lower *K* values, reflecting a more consistent wave climate with less pronounced deviations from the average wave conditions.

These findings underscore that wave climate alone is insufficient to define a local Peak Over Threshold (POT) accounting for the severity of storm events affecting the coastline. Instead, regional sensitivity emerges from the interplay between storm frequency, storm intensity, and the deviation from mean wave climate. To integrate these components, we introduce the Coastal Storm Sensitivity (CSS) index:

1 $$\begin{aligned} CSS = \frac{H_s^{\text {storm}}}{\overline{H_s}} \times NS \end{aligned}$$

In Eq. 1, $H_s^{\text {storm}}$ is the offshore significant storm wave height derived from the storm composites (SCs) (see Methods); $\overline{H_s}$ is the mean offshore significant wave height (1958–2018); and NS denotes the storm number at each location, defined as the ratio of the total number of storms to the number of years.

**Fig. 1:**
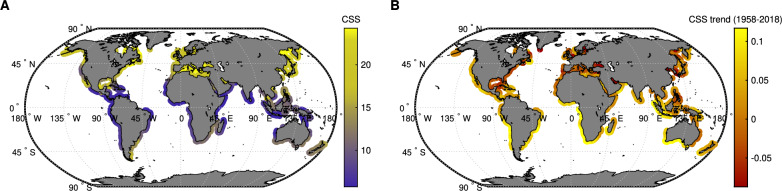
Global distribution of the Coastal Storm Sensitivity index and its associated trend. (**A**) Average CSS values for the period 1958–2018 across the world’s coastlines, highlighting regions more prone to significant storm impacts. (**B**) CSS trend (yr$^{-1}$) between 1958 to 2018, showing a net positive trend along west-facing coasts within the Southern Hemisphere’s primary storm tracks, and a net negative trend in semi-enclosed seas. The world maps were generated using MATLAB R2023b (https://matlab.mathworks.com).

Globally, 14 $\%$ of coastlines exhibit low sensitivity (CSS $\le$ 10) with infrequent storms and minimal disequilibrium (Fig. 1.A). Yet high-storm-frequency regions display divergent behaviors: while semi-enclosed seas exhibit high CSS values, open coasts like the NAWC and WE show lower CSS despite greater storm wave energy (SM, Fig SM4), consistent with the patterns previously indicated by skewness and kurtosis metrics (see SM, Fig. SM13). Long-term trends (1958-2018) indicate decreasing CSS across semi-enclosed seas and the North American East Coast, while positive trends emerge along coasts in the Southern Hemisphere’s primary storm tracks (Fig. 1.B). These shifts are consistent with documented increases in mean wave heights across the eastern tropical South Pacific and the Southern Ocean^39^. Trend decomposition (SM, Fig. SM18) reveals that increasing storm frequency is the primary driver of positive CSS trends whereas rising $\overline{H_s}$ exert a dampening effect by reducing the relative contrast between storm and background conditions. Declining CSS is thus largely attributed to a combination of rising $\overline{H_s}$ and decreasing storm occurrence, even when storm wave heights slightly increase.

While CSS offers a useful proxy for potential storm impact, the relationship between hydrodynamic drivers and shoreline response remains non-linear and highly context-dependent $^{?,}$^40,41^. Beach state, inherited morphology and sediment budget conditions modulate how coastlines absorb or amplify storm impacts^6,7^. Storms should therefore be conceptualized as compound events^42^, wherein hydrodynamic and morphodynamic interact in shaping their net imprint on the coast.

### Shoreline response to storm climates

Storm wave generation occurs at spatial scales that naturally lend themselves to regional analysis. In subtropical latitudes, extratropical cyclones (ETCs), driven by Rossby wave dynamics^43,44^, generate large-scale storm wave fields extending over synoptic scales ($\sim$1000 km). Within the intertropical belt, storm waves are instead driven by mesoscale processes such as tropical cyclones (TCs), with fetches ranging from tens to hundreds of kilometers^45^. These differences in forcing scales underpin regional contrasts in shoreline response.

**Fig. 2:**
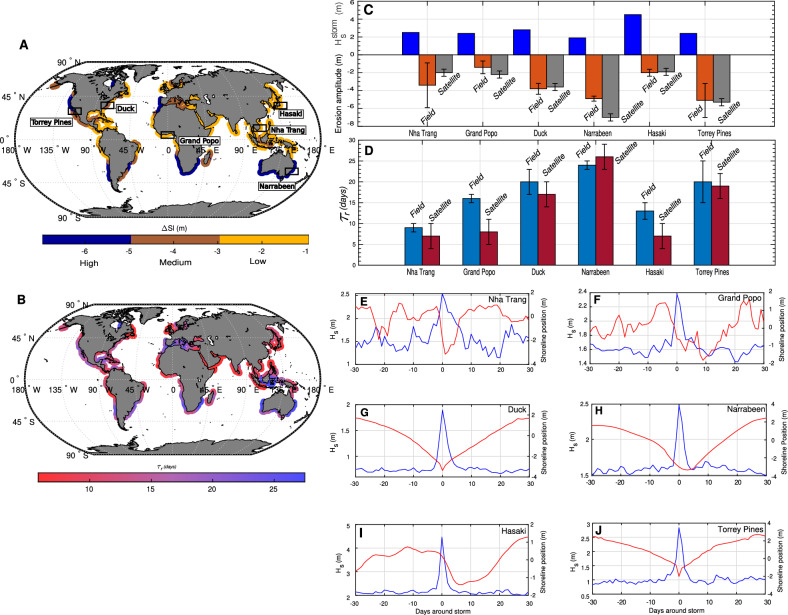
Global storm-induced SDS erosion and post-storm recovery for the period 1993-2016, with *in situ* **comparison.** (**A**) Global distribution of storm-induced shoreline erosion ($\Delta Sl$, in meters), highlighting regional contrasts in land loss. (**B**) Global distribution of SDS post-storm recovery times ($\tau _r$, in days), derived from Eq. 2. (**C**–**D**) Comparison of $\Delta Sl$ and $\tau _r$ estimates from satellite-derived and *in situ* data. Corresponding values and error bars are reported in Table 1. Uncertainties for the *in situ* data were derived by generating SCs while segregating storms by duration and clustering (see SM, Fig. SM9-12). For the satellite-derived data, uncertainties were estimated using synthetic SCs that mimic the satellite temporal resolution, providing a best-guess approximation of the associated error (see Methods). (**E**–**F**) Storm composites at the six *in situ* sites, showing both the hydrodynamic storm signal (solid blue) and the associated shoreline response (solid red). $R^2$ and RMSE values comparing satellite-derived and *in situ* storm composites are also provided in Table 1. The world maps were generated using MATLAB R2023b (https://matlab.mathworks.com).

Here, we quantify these responses using daily-resolution storm composites (SCs) that pair offshore significant wave height with SDS signals to provide broad-scale climatologies of storm impacts (Methods). These SCs should be interpreted as regional statistical tendencies rather than proxies for the site-specific dynamics of individual beaches. In the absence of widespread regional ground truth, their evaluation is necessarily limited to comparisons with single-site observations. For this study, validation was performed against six ground-based sites deliberately selected based on both methodological and physical criteria. All sites are microtidal, ensuring consistency with the assumptions underlying the satellite-derived shoreline processing. In addition, the sites span a wide range of climatic and morphodynamic contexts, from intertropical to extratropical regions, and encompass contrasting wave climates and coastal settings across multiple continents and coastal orientations (Fig. 2.C–J; Table 1).

Within Fig. 2.A, global SCs highlight coherent patterns of storm-induced shoreline erosion ($\Delta Sl$), with moderate to high amplitudes (–3 to –7 m) along major storm corridors such as Angola–Namibia and Chile, and smaller responses (–1 to –3 m) in the intertropical belt, consistent with its lower CSS index values (Fig. 1.A). Beyond erosion, SCs reveal a positive log–log relationship between mean post-storm wave energy ($E_w$) and shoreline recovery rates (see SM, Fig. SM15). Assuming recovery rates remain approximately constant throughout the process (a simplification, as beach state and wave climate variability can introduce deviations), this relationship allows estimation of a regional post-storm recovery time ($\tau _r$, in days):

2 $$\begin{aligned} \tau _r = \frac{\mid \Delta Sl \mid }{aE_w^k} \end{aligned}$$

where $\Delta Sl$ denotes storm-induced shoreline retreat (m), $E_w$ is calculated as $E_w = \overline{H_s}^2 \rho g/16$, and *a* and *k* are empirical coefficients (0.0142 and 0.35, respectively; see Methods).

The resulting patterns (Fig. 2.B) suggest relatively rapid recovery across the intertropical band, with $\tau _r$ typically below 15 days, while subtropical regions exhibit more variable responses (15–30 days), reflecting differences in both erosion magnitude and post-storm wave energy. Comparison with *in situ* estimates indicates that inter-site variability is reasonably captured (R$^{2}$ = 0.74; Fig. 13).

These regional-scale patterns provide a first global benchmark for evaluating sandy shoreline resilience. While they cannot substitute for detailed local analyses, they offer valuable context for assessing whether coastlines are able to re-equilibrate between successive storm events; a key determinant of their long-term morphological stability under changing storm climates.

### Storm-induced beach tipping point

Field observations and laboratory experiments suggest that storm sequencing can amplify beach erosion when inter-storm recovery remains incomplete^21,40,46–48^, highlighting the need to assess beach sensitivity to storm frequency. This non-linear behavior has been conceptualised in shoreline models as a form of “beach memory,” in which recovery lags behind rapid fluctuations in wave forcing^49^.

While projections indicate that tropical cyclones (TCs) may intensify under warming, long-term trends in extratropical cyclone (ETC) wind fields remain uncertain^9^. Nevertheless, offshore significant wave height time series derived from historical records and reanalyses provide an empirical basis for exploring how the frequency of storm events has evolved. From these data, we estimate inter-storm intervals worldwide ($\Delta t_s$, in days) and compare them with regional recovery times ($\tau _r$) derived previously.

**Fig. 3:**
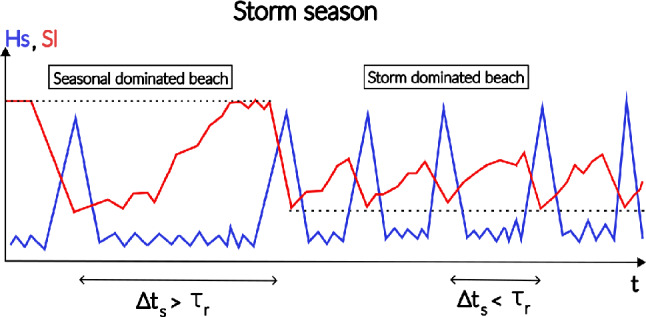
Conceptual scheme of the storm-dominated regime. The scheme illustrates the conceptual behavior of a sandy beach shoreline (solid red) transitioning from seasonal to storm dominance as a tipping point is crossed ($\Delta t_s/\tau _r<$ 1) driven by an increase in storm wave frequency (solid blue line).

We propose a tipping-point framework based on the ratio $\Delta t_s/\tau _r$, which explicitly accounts for the sequencing of storm events relative to shoreline recovery (conceptualized in Fig. 3). Shoreline recovery time $\tau _r$ is estimated from statistically robust storm composites and represents a characteristic post-storm re-equilibration timescale that implicitly integrates all storm occurrence patterns, including closely spaced or back-to-back storm events. Independently, inter-storm intervals $\Delta t_s$ are derived from long-term wave climatology.

When $\Delta t_s/\tau _r < 1$, successive storms occur more rapidly than the shoreline can recover, implying that storm impacts systematically overlap in time and that shoreline evolution is conditioned by incomplete recovery from previous events. This regime corresponds to storm-dominated behavior, where cumulative impacts and persistence of storm signatures are expected. Conversely, when $\Delta t_s/\tau _r > 1$, sufficient time exists between storms for recovery, and shoreline evolution remains primarily seasonally controlled. Because storm composites do not resolve individual event sequencing but instead aggregate a large number of events to ensure statistical robustness, this framework is intended as a climatological indicator rather than a deterministic predictor of event-scale shoreline response.

**Fig. 4:**
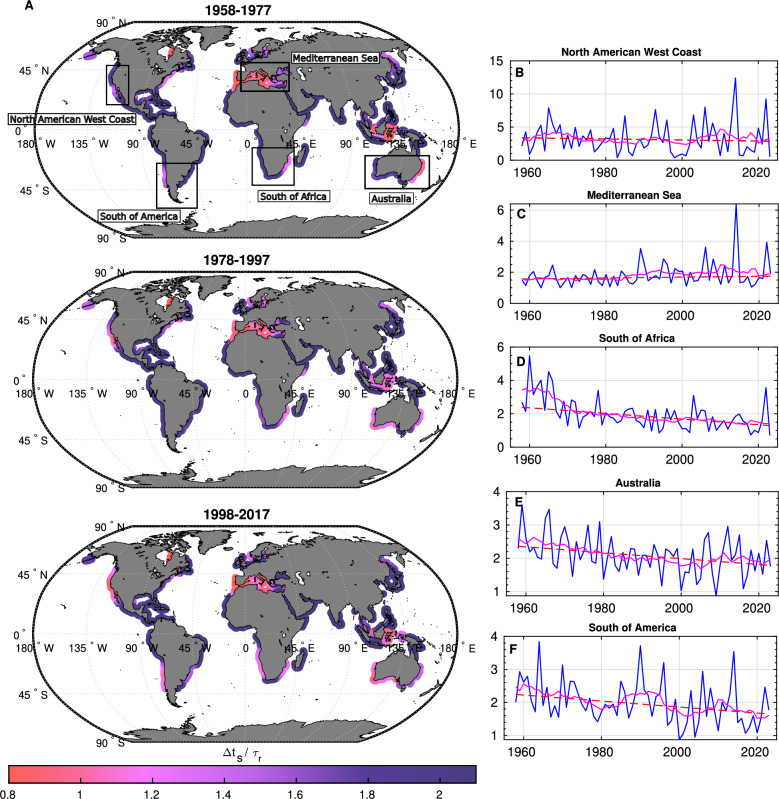
Climatology of the tipping point $\Delta t_s/\tau _r$ over 60 years along with a focus on regional trends. (**A**) 20-year periods average of $\Delta t_s/\tau _r$ presenting regional hotspots of the tipping point evolution. (**B**-**F**) Long term regional trends of $\Delta t_s/\tau _r$ for areas flagged in (**A**). Every zone, except the Mediterranean, show a negative trend, implying increasing pressure on beach resilience due to storms. All trends successfully passed the Mann-Kendall test (Methods). Note that the long-term evolution of this tipping point is also seasonally modulated at the regional scale. More details can be found in the Supplementary Material, in Fig. SM19. The world maps were generated using MATLAB R2023b (https://matlab.mathworks.com).

**Fig. 5:**
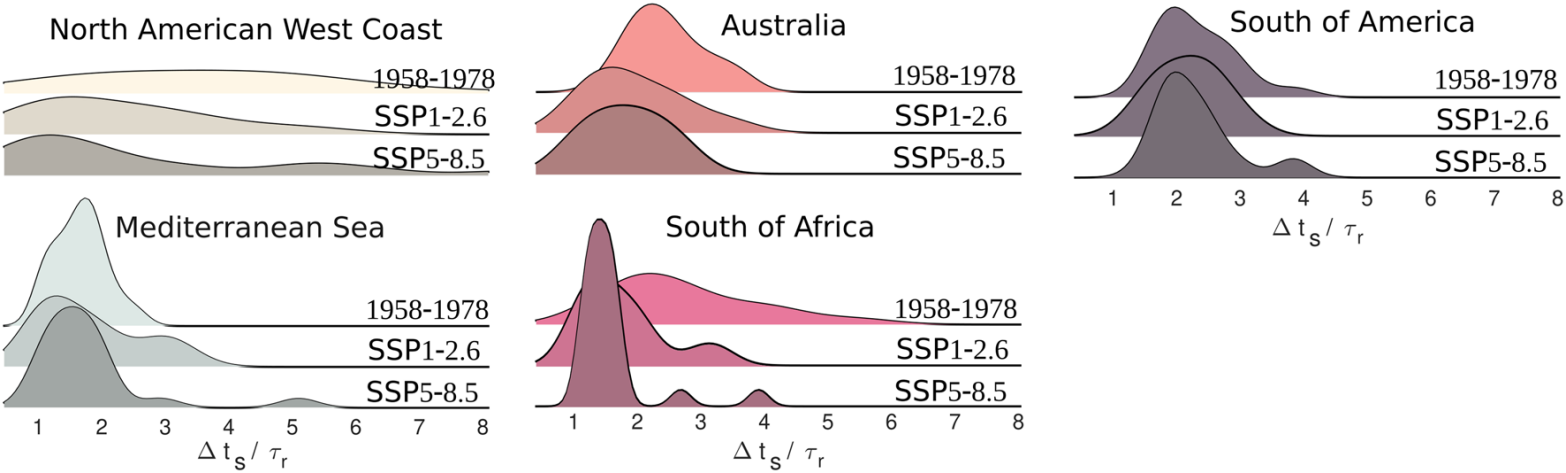
Tipping point distribution evolution toward 2100’s horizon in storm-dominated hot-spots. The tipping point was computed using similar present-day $\tau _r$ values and a 20-year climatology (2080–2100) of $\Delta t_s$ derived from the CMIP6 GCM global wind-wave climate ensemble forced by the IPCC “best-case” (SSP1-2.6) and “worst-case” (SSP5-8.5) scenarios^50^. Comparison with the tipping-point climatology over the period 1958–1978 shows that all areas of interest exhibit a shift in distribution toward more storm-dominated prone values (i.e., shifting toward 1 and below). Y-axis corresponds to the number of locations.

A two-decades climatology of this ratio over 60 years reveals an increase in storm-dominated conditions across several regional hotspots (Fig. 4). Between 1958–1977 and 1998–2017, approximately 2% of the beaches in our dataset (about 1400 km of coast) shifted from seasonal to storm-dominated regimes, notably along the western margins of the Americas, parts of Southeast Asia, and enclosed basins. These shifts correspond to decreasing trends in $\Delta t_s/\tau _r$ (except in the Mediterranean), consistent with shorter inter-storm intervals ($\Delta t_s$ decreased globally by 27%) and increased storm counts (NS rose by 16%). Some regions, such as South America and Indonesia, exhibit decadal-scale modulations, likely reflecting additional climatic or oceanographic controls. The Mediterranean remains the region with the largest cumulative extent of storm-dominated shoreline ($\sim$5300 km), despite showing a weakly positive trend in $\Delta t_s$, in line with the CSS negative trend observed in Fig. 1.B. To explore future trajectories, we used the CMIP6 global wind-wave climate ensemble^50^, under both low-emission (SSP1-2.6) and high-emission (SSP5-8.5) scenarios. Assuming present-day recovery rates remain constant (a simplification given the potential for human intervention and evolving coastal processes) we estimated $\Delta t_s$ over 2080–2100 to project the distribution of tipping-point ratios in current hotspots (Fig. 5). Results suggest further increases in storm dominance across several regions, with shifts in the entire $\Delta t_s/\tau _r$ distribution toward values near or below one. This implies a heightened risk of storm clustering overwhelming natural recovery cycles, although the precise local manifestation will depend on site-specific morphodynamics and management.

Taken together, this framework provides a first-order global indicator of where storm sequencing may outpace natural recovery. While subject to uncertainties in coarse shoreline datasets and empirical recovery models, it offers a complementary perspective to local analyses, highlighting regions where more detailed field-based and high-resolution satellite studies are most urgently needed.

### Limitations

Uncertainties in SDS position datasets remain substantial, making comparison with *in situ* measurements essential (Methods). Site-specific beach surveys remain the most reliable means to capture local, fine-scale reality^51,52^. Yet, such monitoring is inherently limited in both spatial extent and temporal duration, and may therefore represent only a fraction of the full spectrum of beach dynamics^53^. This limitation is especially critical given that most sandy coastlines remain undocumented, primarily because of the cost and logistical effort of continuous field measurements.

In this context, satellite-based observations provide a powerful complement: by widening spatial coverage, extending observation periods, and encompassing a broad diversity of coastal environments, regional to global products offer insights that are otherwise unattainable^32–34,54^. Regionally aggregated studies (>100–1000 km) can be used to assess the climatology of common beach behaviors, even though they inevitably smooth out local diversity. This distinction mirrors meteorological sciences: analyzing the climatology of a variable over a given region does not preclude the occurrence of local anomalies at finer temporal and spatial scales. The same reasoning applies in coastal science: *in situ* datasets provide the detail required to uncover processes and mechanisms unresolved at broader scales, while satellite observations reveal the baseline trends and regional coherence across thousands of kilometers of shoreline.

Although the philosophical debate between local and global perspectives has been addressed by Almar et al. ^55^, it is worth emphasizing that the present dataset (the only publicly available global product at monthly resolution, produced in 2020) covers two decades at a native resolution of about $0.27^\circ$ in latitude, aggregated over a $3^\circ$ radius, thereby making it directly suitable for deriving regional climatologies. Comparison with single-site surveys is therefore not the most appropriate benchmark. While it is encouraging that the dataset can still capture aspects of local dynamics (albeit blended with neighboring signals), a more robust evaluation should involve comparing SDS-based climatologies against spatially averaged *in situ* observations and analyzing the emerging trends. In practice, this validation remains challenging and is currently only possible indirectly through a cascade approach, where regionally averaged datasets are compared with higher-resolution satellite products, themselves validated against sparse *in situ* measurements. We applied this framework along the western U.S. coast for SC climatologies, and the results statistically confirm the validity of our approach (Methods). A similar multi-scale comparison was recently performed by Almar et al. (in review) directly on SDS time-series at both fine (200 m) and coarse ($\sim$ 25 km) resolutions, demonstrating that even coarse-resolution products can reproduce seasonal-to-interannual shoreline variability across regional ($\sim$ 600 km) scales (Fig. SM20). Altogether, this global exploration of shoreline dynamics, together with the identification of regional patterns and hotspots, underscores the complementarity between satellite observations and targeted in situ surveys. Strengthening survey efforts in so-called “remote areas,” particularly within the intertropical belt and the Global South, will be crucial to unravel the physical mechanisms driving coastal variability in these important yet still under-documented regions.

A further limitation concerns the seasonal dependence of storm-driven shoreline response. Shoreline erosion and recovery are known to depend strongly on the pre-storm beach state and sedimentary configuration, which are themselves seasonally modulated. Thus, while storms may exhibit comparable hydrodynamic forcing, separating them according to their season of occurrence in order to resolve seasonal differences in recovery time is not operationally feasible with the current satellite shoreline record. The primary constraint is the still limited temporal extent of global satellite-derived shoreline datasets, which restricts the number of storm events available for stratified analyses and would lead to a significant loss of statistical robustness. Addressing this limitation will require longer shoreline time series, which should ultimately allow the construction of seasonally resolved storm composites and a more explicit assessment of the role of beach state in post-storm recovery. In the present analysis, this seasonal control is not treated explicitly. Instead, it is implicitly incorporated through the aggregation of a large number of storm events occurring across different seasons. The objective is therefore to characterize a statistically robust mean storm response and recovery behavior, rather than season-specific dynamics. The reliability of the composite approach is intrinsically linked to the number of events available for aggregation.

### Discussion

The detection of a potential storm-dominated tipping point raises important questions about the cumulative impacts of clustered extreme events on the long-term evolution of sandy beaches. Field observations have shown that recovery after intense storm seasons may remain incomplete^14,56^, suggesting that repeated exposure can leave a legacy effect on shoreline position. In our monthly-scale and regional to global analysis, this behaviour emerges statistically as a shift towards storm-imposed equilibrium during storm seasons. This may delay or suppress subsequent recovery. While the strength of this signal is likely to vary depending on local morphology, sediment supply and management, its repetition could contribute to longer-term erosional trends.

One way to tentatively explore this cumulative signal is to compare shoreline position trends during storm seasons with those observed several months later, when seasonal recovery is typically expected. Similar slopes in both trends would indicate that long-term drivers (e.g. sea-level rise, sediment imbalance and anthropogenic pressure) may dominate. Conversely, if the post-storm trend remains negative and diverges from the storm-season trend, this suggests that storm clustering is an additional factor contributing to persistent shoreline recession. However, we stress that such diagnostics are only first-order indicators at regional scales and should not be interpreted as direct evidence of local shoreline response.

These considerations are particularly relevant in the context of climate change, where storm intensity and frequency are expected to increase alongside rising sea levels. The tipping-point framework introduced here should be viewed as a tool for identifying large-scale statistical trends, rather than providing local, deterministic forecasts. It is valuable for highlighting potential regional hotspots and generating hypotheses that can be tested using higher-resolution satellite products and *in situ* surveys. Future work should focus on developing early-warning indicators of tipping behavior, as well as quantifying morphodynamic thresholds beyond which natural recovery becomes impaired. Strengthening the complementarity between global satellite observations and local ground-truthing is essential for assessing the robustness and practical relevance of these signals.

## Conclusion

Using two decades of satellite-derived shoreline observations combined with six decades of wave reanalysis, this study provides a global assessment of how microtidal sandy beach responses to storm climates manifest at regional scales. By statistically resolving storm-induced erosion and post-storm recovery at synoptic scales, we show that monthly satellite datasets can retain the imprint of sub-seasonal storm dynamics when aggregated appropriately.

We introduce a first-order, transferable statistical framework based on the ratio between inter-storm intervals and shoreline recovery times, which diagnoses a transition from seasonally controlled to storm-dominated shoreline regimes. Since the late 1950s, this transition has affected an increasing fraction of the global coastline, with coherent regional hotspots emerging across multiple basins. While this framework is not intended to predict site-specific behavior, it provides a large-scale diagnostic of where storm sequencing is most likely to outpace natural recovery. By placing local observations within a global context, it offers a quantitative basis for targeting future high-resolution satellite analyses and field-based investigations. As storm climates continue to evolve, accounting for storm-driven regime shifts will be essential for anticipating long-term shoreline trajectories.

## Methods

### Wave dataset

Offshore wave conditions were characterized using the ERA5 reanalysis, a globally consistent dataset produced by the European Center for Medium-Range Weather Forecasts^57^. Hourly time series of significant wave heights, spanning 60-years period (1958-2018), were spatially and temporally interpolated to match the grid and resolution of the SDS dataset. Daily means were computed to align with the temporal scale used in storm composite construction. It should be noted that ERA5 wave reanalysis may exhibit region-dependent biases, which can introduce uncertainties in inter-regional comparisons. However, the reliability of the ERA5 wave hindcast has been extensively assessed against in situ buoy records across a wide range of ocean basins and coastal settings, with detailed global and regional performance metrics provided in the Supplementary Material of Lobeto et al.^38^.

### Shoreline dataset

The shoreline dataset underpinning this study spans over approximately 1.5 million kilometers along the global coastline, sampled at a mean spatial resolution of $0.27^\circ$ (equivalent to 27 km). Following the approach of Almar et al.^22^, monthly shoreline composites were extracted from 1993 to 2016 using SDS, based on the Normalized Difference Water Index (NDWI) applied to Landsat 5, 7 and 8 imagery via Google Earth Engine. These composites rely on the waterline proxy, defined as the instantaneous intersection of land and water surfaces, providing a consistent, remotely sensed signal of shoreline position. Although widely used, this method is not without limitations. Notably, the lack of tidal correction in SDS introduces positional uncertainties in meso to macrotidal settings, potentially obscuring storm-driven signals^37,58^. To mitigate such biases, we restricted the analysis to microtidal coastlines, where SDS-associated errors remain within an acceptable range of approximately 5 to 10 m^51,59^. This filtering ensures that observed shoreline changes reflect genuine morphodynamic responses rather than artifacts of tidal variability.

**Fig. 6:**
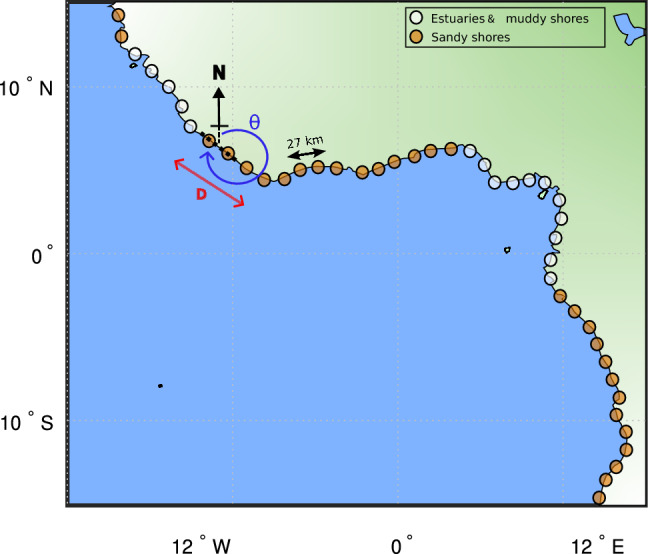
SDS dataset pre-processing schematics. The map illustrates the metrics used to merge transects based on the bearing angle ($\theta$) of two adjacent grid points. Distances between grid points are for illustrative purposes only.

To further refine the dataset toward dynamic sandy coasts, a multi-step spatial filter was applied. Estuaries and sheltered low-energy regions were excluded using a spatial density mask, and muddy shorelines, accounting for roughly 15$\%$ of the global coastline, were filtered out based on the classification criteria of Hulskamp et al.^24^ (SM, Fig. SM14). This ensure the dataset reflects primarily open and embayed, wave dominated sandy beaches. To improve signal robustness and allow for the assessment of regional patterns, shoreline time series were aggregated spatially following a methodology adapted by Castelle et al.^60^. Transects were grouped according to their coastal orientation and proximity, computed using the bearing angle ($\theta$) between adjacent sites averaged within a $D = 500$ km radius. A threshold range of $\delta \theta = \pm 15^\circ$ was applied to merge locations with similar wave exposure (Fig. 6). Finally, each SDS time series was linearly resampled to a daily timestep and detrended from its seasonal climatology, resulting in a refined dataset suitable for the generation of storm morphological imprint climatologies across approximately $7.4 \times 10^4$ km of sandy coastline.

### Storm wave detection

Detecting storm waves at the global scale presents unique challenges due to the vast heterogeneity of local wave climates. While the Peak Over Threshold (POT) method is commonly used in coastal storm analysis^16,61,62^, its application at the global scale is limited. This is because storm impacts are not defined solely by absolute wave heights, but rather by their deviation from local wave conditions, which influence their potential impact for morphological change^63^. To overcome this, we employed a relative, statistically-framed threshold to define storm events based on their deviation from the median of the wave height distribution as: $\tilde{H_s} + \alpha \sigma< H_{s}< \tilde{H_s} + \gamma \sigma$. Where $\tilde{H_s}$ is the offshore significant wave height median (m) and $\sigma$ the standard deviation of the distribution (m). $\alpha$ and $\gamma$ have been empirically determined to encompass the central range of impactful storm events, dynamically spanning the $95^{th}$ to $99^{th}$ percentiles of the distributions, with values of 2.8 and 5, respectively. This thresholding strategy offers two key advantages. First, it accounts for regional variability in wave climate, ensuring that storm identification is locally meaningful. Second, it excludes the most extreme events, often associated with atypical morphological impacts (return periods of years to decades). Importantly, this method does not require a predefined storm duration, facilitating its integration with daily-aggregated wave data. The robustness of this approach is supported by the close agreement between the resulting global storm counts and those reported by Lobeto et al.^38^, who used a fixed $95^{th}$ percentile threshold to define storm events (see Supplementary Material for additional details on the characterization of storm wave spatial distribution, seasonality, and duration in Figs. SM1–SM5).

### Storm composite generation

#### Storm wave aggregates

**Fig. 7:**
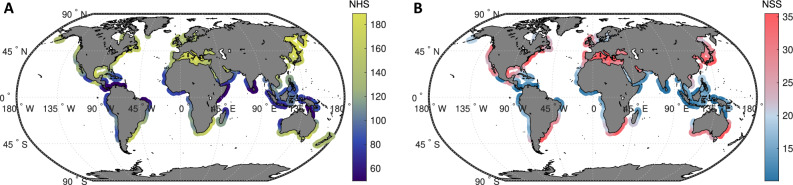
Global distribution of the number of storms aggregated to generate the storm composites. (**A**) Number of Hydrodynamic Signals (NHS) aggregated per site, with fewer storms flagged in the intertropical band. (**B**) Number of Shoreline Signals (NSS) aggregated per site after applying the selection criteria shown in Fig. 8. The world maps were generated using MATLAB R2023b (https://matlab.mathworks.com).

For each storm event identified, a 60-day time series of offshore significant wave height, centered on the storm peak, is extracted, yielding a detailed record of pre-storm, peak, and post-storm wave conditions. This process results in a rich catalog of storm wave signals at each location. On average, 128 events are retained per location, with some sites encompassing up to 248 storms events over the study period (Fig. 7.A). These individual time series are then composited to generate an averaged storm wave signal that reflects the characteristic morphology and intensity of storm forcing at each site. The aggregation of numerous storm events enhances the statistical robustness of the composite, smoothing local anomalies while preserving the storm dynamics.

**Fig. 8:**
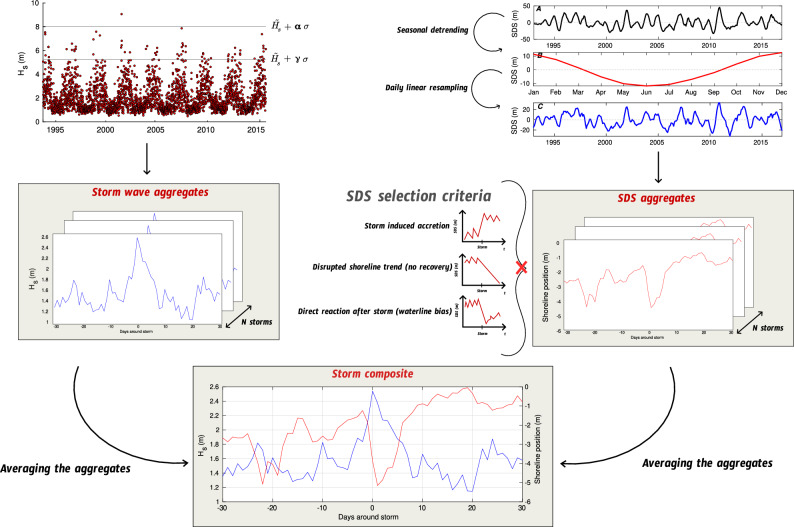
Storm composite generation algorithm schematic. Whenever a storm wave is detected by the algorithm, a 60-day storm-centered time series of offshore significant wave height is stored, enabling the creation of a storm wave signal collection at each location. Similarly to the storm wave aggregates, 60-day windowed time series of daily SDS are generated for each flagged storm. The time series compiled in the SDS aggregates are then filtered based on selection criteria before being averaged, ensuring that only those exhibiting both storm-induced SDS erosion and post-storm SDS recovery within the time window are retained. The final integration of storm wave and SDS aggregates produces the storm composites.

#### SDS aggregates

Mirroring the storm wave aggregates, 60-day windowed time series of daily SDS positions, centered on each flagged storm event, are compiled to assess typical shoreline responses at each location. However, due to the inherently more complex and variable nature of shoreline behavior, a rigorous filtering process is required before composite generation. Several factors motivate this selective approach. First, storm-induced accretion may occur under specific conditions, such as the erosion of dunes supplying sediment to the beachface^8,64^ or by aeolian processes during offshore wind condition^65,66^, leading to misleading positive shoreline displacements. Second, the SDS metric based on the waterline proxy is sensitive to the instantaneous water level variations, particularly following storm events, potentially distorting short-term recovery signals. Third, extreme events may introduce long-lived anomalies in shoreline position, masking the more typical post-storm response patterns^15^. To mitigate these issues, SDS time series are filtered using a set of conservative criteria (Fig. 8). Events displaying shoreline advance rather than erosion during storm peak, as well as those with disturbances persisting beyond 30 days, are excluded. In addition, signals exhibiting an apparent shoreline response within 24 hours of the storm peak are discarded to reduce potential bias from transient waterline fluctuation unrelated to sediment redistribution. Following this stringent selection, an average of 25 SDS time series per site are retained to generate composite shoreline responses of both storm-induced erosion and subsequent recovery within the 60-day analysis window (Fig. 7.B).

### Super resolution: proof of concept

**Fig. 9:**
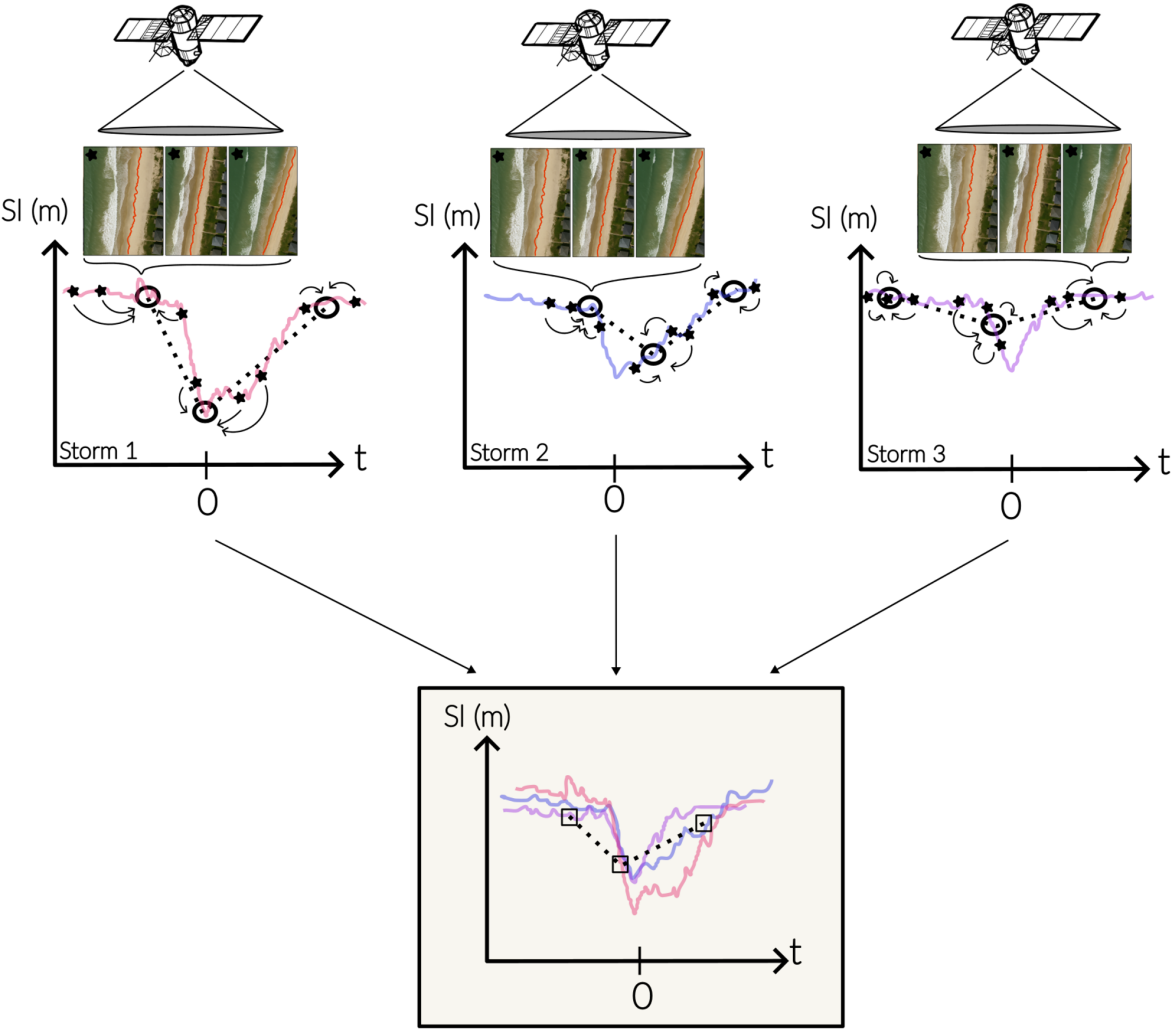
Conceptual illustration of storm composite inferring from shoreline observations. Stars indicate shoreline positions at each satellite revisit. Circles represent the average of three revisits, used as a monthly estimate. Black dashed lines show reconstructed trajectories derived from these averages, while the colored curves correspond to the actual shoreline evolution in real time (not fully captured by the satellite). In the lower panel, squares mark the mean of the three reconstructed trajectories, illustrating the statistical superposition process leading to the storm composite. The robustness of the reconstructed storm imprint on shoreline position increase with the number of storm aggregated.

The effectiveness of capturing storm imprints on shorelines using monthly SDS resampled at a daily timescale becomes statistically possible because a monthly SDS position is an aggregate of several shoreline position detections, where the number of aggregates is correlated with satellite revisit frequency and atmospheric disturbances (e.g., clouds, aerosol plumes from wildfires or volcanic activity). Usually, SDS positions are derived from the average of 3 to 4 satellite revisits (sometimes more, depending on the mission). Consequently, each monthly SDS estimate contains the imprint of sub-scale events, such as storm erosion and beach recovery. As conceptually illustrated in Fig. 9, it may become possible to retrieve the imprint of an event (under the assumption that its pattern is quasi-similar from event to event), by compiling a statistically significant number of events flagged through an independent marker (here, ERA5 wind-wave dataset). In the present study, the assumption of similar patterns is addressed by carefully excluding all non-typical erosive-storm patterns before generating the storm composites, as explained in Fig. 9.

### Super resolution: proof of work

**Fig. 10:**
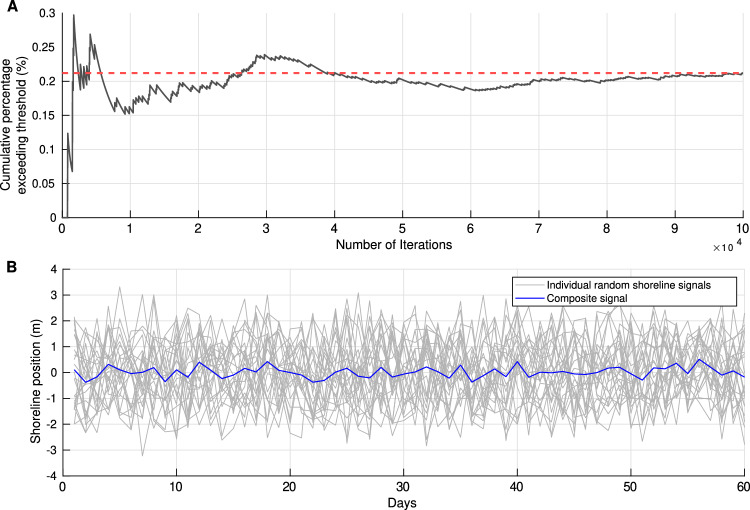
Synthetic shoreline data used to assess the statistical robustness of storm composites. (**A**) Cumulative percentage exceeding the threshold for selection criteria applied to synthetic random shoreline signals. (**B**) Storm composite (solid blue) generated using 25 random synthetic shoreline signals (solid grays), showing that it is unlikely that storm composite selection criteria would generate forced pattern of storm induced erosion and post-storm shoreline recovery pattern from randomly selected shoreline time-window.

Capturing daily to weekly-scale morphodynamic responses from monthly satellite-derived datasets presents a fundamental challenge for coastal monitoring. To evaluate whether the observed storm-related shoreline signals could emerge from random sampling alone, we applied the SDS filtering criteria to 100,000 randomized shoreline time series. This large-scale approach allows the determination of a randomness threshold, that is, the probability that a randomly generated SDS signal would satisfy the filtering criteria by chance (Fig. 10.A). Results show that only 0.21$\%$ of these random signals satisfied the selection process, confirming that the retained SDS aggregates represent statistically robust and morphodynamically meaningful responses. Furthermore, white noise fails to generate a coherent storm composite signal when subjected to the same process (Fig. 10.B).

**Fig. 11:**
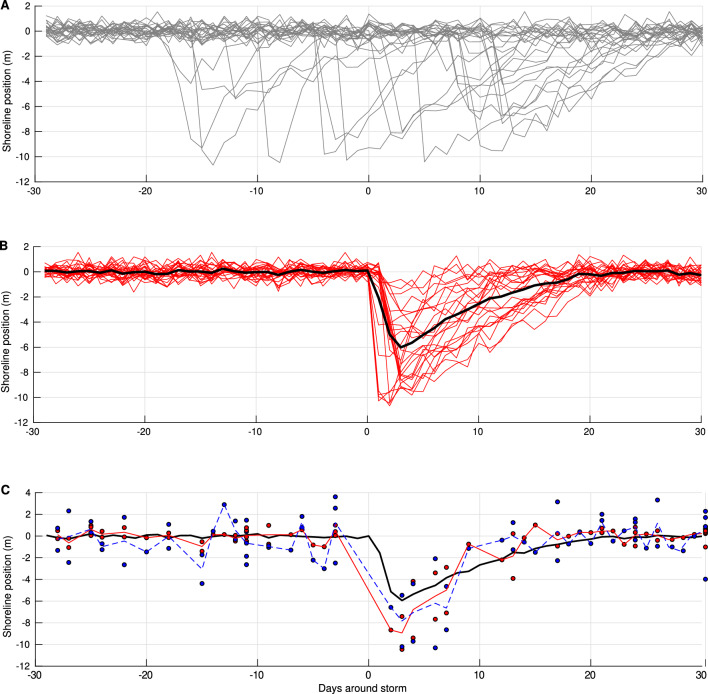
Synthetic shoreline response to storms used to evaluate the ability of monthly observations to resolve sub-seasonal storm driven dynamics. (**A**) Example of 25 daily-resolved synthetic shoreline time series. Each series combines natural day-to-day shoreline variability (normally distributed, $\mu = 0$, $\sigma = 1$ m), synthetic storm duration (uniformly distributed, $\mu = 2$, $\sigma = 0.8$ days), and storm responses including both storm-induced erosion and post-storm recovery phases. Parameters values were selected based on direct observations obtained from storm composites, using both *in situ* and satellite-derived data. (**B**) The same time series, aligned on the storm peak-centered (solid red) and their averaged response (solid black) illustrating the typical shoreline adjustment to storm forcing. (**C**) Composite response from (**B**) (solid black), overlaid with simulated satellite-derived shoreline observations represented by two simulations of monthly under-sampling to replicate SDS composites resulting from satellite acquisitions. The first simulation corresponds to random acquisitions over the 25 synthetic shoreline time series within the time window, assuming no SDS error (red points). The average of these observations (solid red) shows that both erosion and recovery processes are well captured. The second simulation accounts for the well-known microtidal environments SDS associated error distributions^51,59^ (blue points), which are here, $\mu = 0$, $\sigma = 2.5$ m. The average of these observations (dashed blue) confirms that achieving super-resolution—i.e., resolving sub-sampled temporal events—is possible if the event sample is statistically sufficient.

To test whether monthly satellite-derived datasets can nonetheless resolve storm imprint on beach dynamics when aggregated appropriately, a series of synthetic experiments was conducted. Based on the globally averaged number of shoreline signals (NSS), twenty-five synthetic 60-day shoreline time series replicating the structure of actual storm-affected *in situ* and satellite-based time-series were generated. These synthetic signals incorporate: (1) the natural day-to-day shoreline variability following a normal distribution ($\mu = 0$, $\sigma = 1$ m), and the storm durations following a uniform distribution ($\mu = 2$, $\sigma = 0.8$ days); (2) the shoreline response to storms includes both storm-induced erosion and post-storm recovery, modeled using uniform distributions with $\mu = 5.5$, $\sigma = 2.9$ m for erosion, and $\mu = 15$, $\sigma = 6$ m for recovery (Fig. 11.A). By centering the storm peak of each time series within the 60-day window and averaging across the 25 synthetic events, a composite storm response was obtained (Fig. 11.B). This storm composite represent the storm morphological imprint climatology of the synthetic time-series (i.e. the tendency we want to retrieve from the collection of storm events using satellite imagery). To mimic satellite sampling limitations, these time series were undersampled at monthly resolution, with random timing of acquisitions, yielding 2 to 3 SDS positions per event, analogous to the temporal sparsity of actual SDS products. When overlaid on the high-resolution synthetic composite, this undersampled dataset preserved key features such as the erosion amplitude and recovery trajectory, even when accounting for typical SDS retrieval errors in microtidal environments^51^(Fig. 11.C). One observes that the storm-induced erosion amplitudes assessed in this study fall within this error range. However, the error estimate for a single SDS position estimation and the error estimate resulting from a statistical aggregate of events, which decreases with its size, should not be confused with each other.

**Fig. 12:**
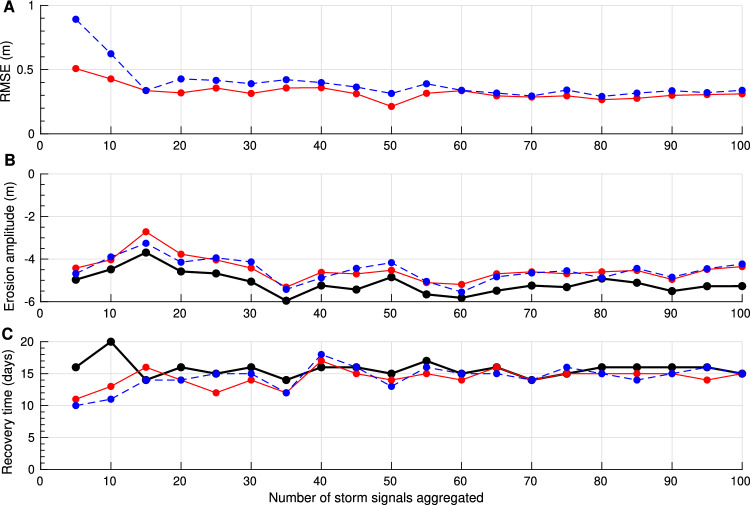
Satellite derived storm composite error assessment on synthetic data depending on the NSS. (**A**) Evolution of the RMSE between the averaged signal (solid black) and the satellite-simulated ones, with (solid blue) and without (solid red) error associated to SDS monitoring. (**B**) Evolution of the amplitude of erosion estimation derived from the average signal (used as a reference) and the ones derived from the satellite-simulated ones. (**C**) Same as (**B**) but for the recovery time.

To further assess the sensitivity of sample size on the quality of the composite, the simulation was repeated for increasing values of NSS (5 to 100, in increments of 5). Results show a rapid convergence of RMSE toward 0.4 m when more than 15 shoreline signals are aggregated, with reliable estimates of erosion and recovery achieved from this threshold onward (Fig. 12). Based on this relationship, a confidence index (CI) is defined to evaluate the robustness of storm composites at each location:

3 $$\begin{aligned} CI = 100 \cdot \frac{NSS_i }{(\overline{NSS} + \sigma _{NSS})} \end{aligned}$$

In Eq. 3, *NSS* represents the worldwide total number of morphological signals retained to generate the storm composite, $NSS_i$ refers to the number of morphological signals retained for the storm composite at a specific location and $\sigma$ is the standard deviation of *NSS*. CI shows high statistical confidence in semi-enclosed sea regions and west facing subtropical coast with high storm frequencies, while the lowest confidence values are observed in the intertropical band (see SM, Fig. SM6).

### Storm induced erosion and post-storm recovery definition

In this study, storm-induced shoreline erosion is defined as $\Delta Sl = (\overline{Sl} + \sigma _{Sl}) - Sl_{min}$ where Sl (m) denotes the shoreline position within the storm composite, $\overline{Sl}$ the average shoreline position within the storm composite window, $\sigma _{Sl}$ the standard deviation of the shoreline and $Sl_{min}$ the minimum of the shoreline signal flagged within the window. Similarly, for *in situ* sites, post-storm recovery time ($\tau _r$, in days) is defined as the time interval between $Sl_{min}$ and the moment at which Sl returns to or exceeds the threshold *Sl* = ($\overline{Sl}$ + $\sigma _{Sl}$), ensuring consistency in quantifying recovery.

### Shoreline recovery rate empirical law

**Fig. 13:**
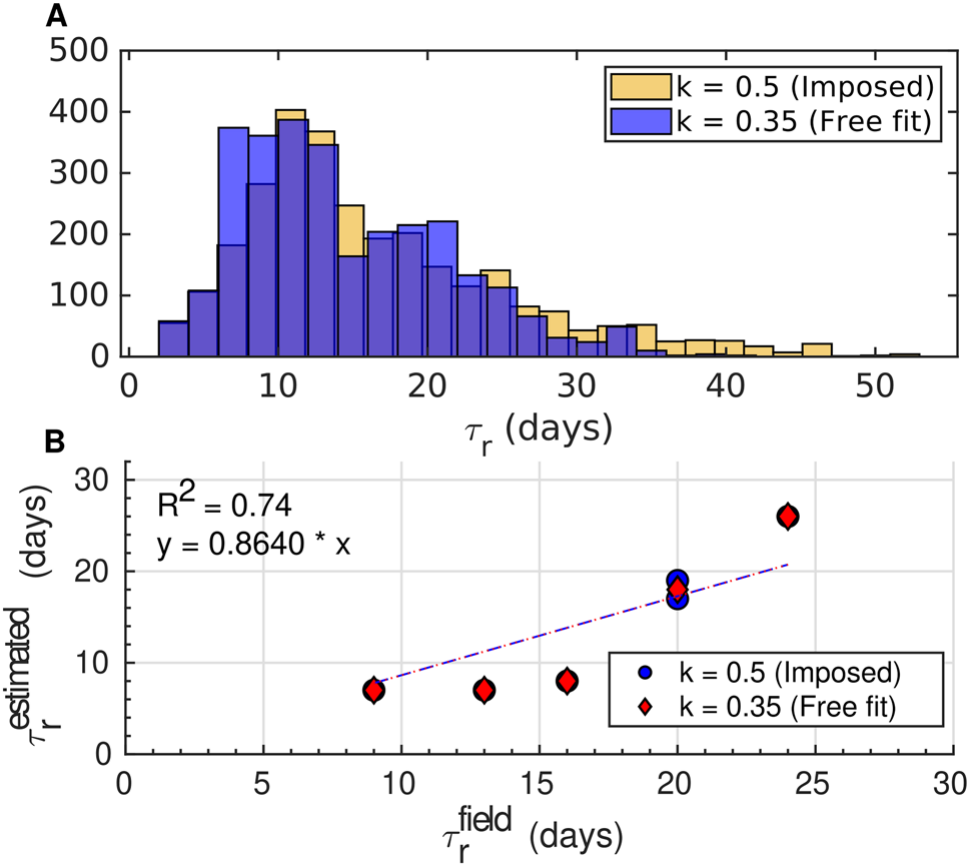
Recovery time distribution and its inter-site variability depending on the value of the parameter *k*. (**A**) Recovery time distribution estimation with (yellow) and without (blue) imposing the slope value of the fit in the log-log space between the post-storm shoreline recovery speed and the average wave energy beach exposure. The two approaches (k=0.5 imposed vs. k=0.35 free fit) yield broadly consistent distributions. (**B**) Inter-site variability captured by the empirical law deduced in this study. The figure compares the response times measured in situ (x-axis) with the values obtained from the empirical law derived in this study (y-axis). Red diamonds represent estimates with a fixed coefficient (k=0.5), while blue dots correspond to the free fit (k=0.35). The linear regressions (blue and red dashed overlined lines) have a slope of 0.864 and a coefficient of determination $R^2$=0.74, indicating that the model captures the inter-site variability of response times reasonably well.

In the log–log regression framework, the recovery rate (*R*) is expressed as $R = a \, E_w^k$, where both the prefactor *a* and the exponent *k* are estimated from the data. A theoretical value of $k=0.5$ is anticipated on dimensional grounds, since it ensures that *R* scales as a velocity and that the recovery time $\tau _r = \Delta Sl / R$ has the correct dimension of time without requiring additional compensation from *a*. Deviations from this value imply that dimensional consistency is restored only through *a*, which then absorbs the influence of unresolved processes (noise, inter-site variability) not captured by the data-inferred relationship. The free fit yields $k = 0.35$, close to this theoretical expectation, indicating that shoreline recovery time is mostly governed by the balance between shoreline excursion and the available wave energy (i.e. the ratio $\Delta Sl / E_w$). To further assess this interpretation, we repeated the regression with the fixed slope value of $k=0.5$. The resulting post-storm shoreline recovery rates distributions are mostly similar to those obtained with the free fit (Fig. 13), confirming that the inferred dynamics are consistent whether *k* is left free or constrained to its theoretical value. We note, however, that both *a* and *k* may vary across regions, reflecting differences in coastal morphology, sediment characteristics, and local hydrodynamic conditions. Assessing such regional dependence is not feasible with the present global dataset and would require regionally focused, high-resolution observations.

### Trends estimation

Trends estimation was conducted with particular attention to the distributional properties of the data. To mitigate the influence of transient anomalies and ensure robust central tendency capture, we deployed a classical interquartile range (IQR) filter: values falling below the $25^{th}$ percentile and above the $75^{th}$ percentile were excluded prior to trend computation. This approach is justified by the low skewness and limited presence of outliers across the dataset (see SM, Fig. SM16-17), which supports the appropriate use of the IQR method. All trends successfully passed the Mann-Kendall test evaluating monotonicity and significance.

### Validation using a high spatial resolution tide corrected SDS dataset

**Fig. 14:**
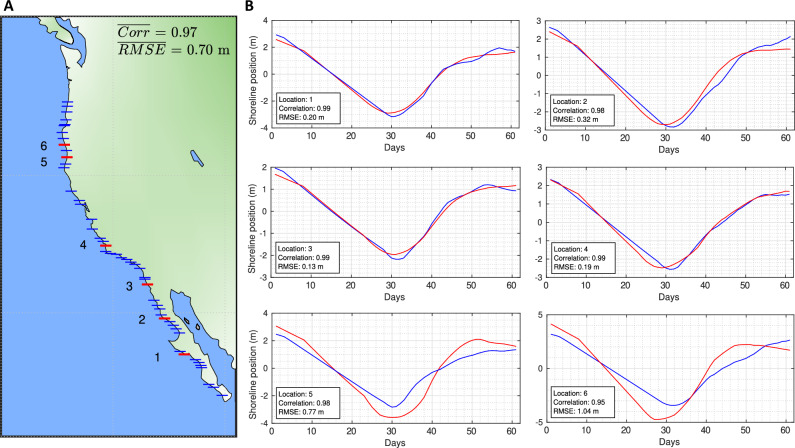
Validation of storm composite signals generated along the west coast of North America between a tide-corrected, high spatial resolution SDS dataset^31^ and the lower spatial resolution dataset used in this study. (**A**) Map of the west coast of North America illustrating the transects used for storm composite signal comparisons, along with the corresponding averaged correlation and RMSE values. Red-labeled transects denote the locations of storm composites presented in subpanel B. (**B**) Storm composite subplots comparing signals derived from the high spatial resolution SDS dataset (solid blue) and the low spatial resolution SDS dataset (solid red) across transects spanning from subtropical to high-latitude regions. The map was generated using MATLAB R2023b (https://matlab.mathworks.com).

To estimate the uncertainties associated with the generation of storm composites from coarse-resolution, non-tide-corrected shoreline data, we performed a comparative validation using a high-resolution (275 m-spaced), tide-corrected shoreline dataset (HRTC) developed by Graffin *et al.*^31^. This dataset spans the western coast of North America and was produced using the Shoreliner extraction toolkit^59^. Tidal corrections were applied using the FES2022 global tide model^67^, and the product has been bench-marked against high-frequency *in situ* measurements at Torrey Pines, yielding performance metrics consistent with state-of-the-art methods^51^. To enable a direct comparison with the coarser dataset used in this study, the HRTC-SDS dataset was first spatially averaged to match our global SDS dataset resolution, and processed using the same storm composite generation methodology. A total of 225 co-located transects, matched within a $\pm 0.025^\circ$ latitude tolerance, were analyzed along the coast. The average correlation between the two storm composite datasets is 0.97, with an average RMSE of 0.70 m across the latitudinal gradient from south to north (Fig. 14).

### Validation with field data

To evaluate the robustness of the satellite-derived storm composites, we compared them against six ground-based coastal monitoring datasets encompassing a broad spectrum of morphodynamic settings and wave climates. These sites include two intertropical microtidal beaches (Nha Trang Bay (Vietnam) and Grand Popo (Benin)), and four subtropical microtidal beaches (Duck Beach and Torrey Pines (USA), Narrabeen (Australia), and Hasaki (Japan)).

Intertropical sites were monitoring continuously over 3.5 years at daily resolution using video-based bathymetry inversion methods^68^, yielding beach profiles, shoreline positions, and significant offshore wave parameters. Validation against field-surveyed profiles showed strong agreement, with RMSEs of 0.14 m at Grand Popo and 0.26 m at Nha Trang. Detailed methodology and accuracy assessments are provided in Thuan et al.^69^ and Abessolo Ondoa et al.^70^.

Subtropical sites offer a range of temporal resolutions and morphodynamic contexts. Duck and Narrabeen beaches benefit from long-term, high-quality shoreline and profile records collected at sub-monthly to monthly intervals, extending over several decades^47,71^, complemented by in situ wave buoy data. Torrey Pines Beach, though lacking concurrent wave measurements, provides consistent shoreline surveys suitable for composite generation^72^. Hasaki Beach is monitored at daily to weekly intervals^73^. ERA-5 reanalysis wave data were used at Torrey Pines and Hasaki beach to identify storm events in the absence of local buoy records.

The storm composite methodology was applied across all six sites to estimate representative storm wave heights and associated shoreline responses. This multi-site validation demonstrates the capacity of the satellite-based approach to capture both erosion and recovery signals under a range of forcing conditions. Table 1 summarizes the number of events retained at each site, along with estimated storm-induced erosion magnitudes, recovery times and validation metrics ($R^2$ and RMSE). The agreement between satellite and field-based storm composites supports the use of this framework for assessing storm impacts on sandy beaches at regional to global scales.

**Table 1:** Field-derived storm composites recap table. Values depicted correspond to the ones plotted in Fig. 2. $\Delta Sl$ and $\tau _r$ where estimated without discriminating storms by their durations or clustering while their uncertainties where derived by generating storm composites when differentiating ’one day’, ’more than one day’, ’single’ and ’cluster’ storms (SM, Fig. SM7-12). $R^2$ and RMSE correspond to the validation metrics between satellite-derived Scs and *in situ* ones. Due to the daily resolution of Nha Trang and Grand Popo, their SCs where undersampled at lower resolution to match the ones of satellite-derived SCs and allow for comparisons.

Sites	Number of storm clustered	Analyzed period	$H_s^{storm}$ (m)	$\Delta Sl$ (m)	$\tau _r$ (days)	$R^2$	RMSE (m)
Nha Trang	22	2013-2016	2.5	-3.4 ± 2.5	9 ± 1	0.71	0.60
Grand Popo	12	2013-2016	2.4	-1.4 ± 0.71	16 ± 1	0.42	0.80
Duck	52	2013-2023	2.8	-3.8 ± 0.61	20 ± 3	0.98	1.40
Narrabeen	221	1979-2014	1.9	-4.9 ± 0.28	24 ± 1	0.88	2.90
Hasaki	190	1986-2016	4.5	-2 ± 0.39	13 ± 2	0.79	0.73
Torrey Pines	221	2003-2016	2.4	-5.1 ± 1.9	20 ± 5	0.74	1.91

## Supplementary Information

Supplementary Information.

## Data Availability

The global sandy satellite-derived shoreline dataset analyzed in this study is accessible via the following link: https://www.pndb.fr/explore/assets/satellite-derived-global-waterline-dataset-with-environmental-forcings-1993-2019/. The ERA-5 wave dataset is available at: https://cds.climate.copernicus.eu/datasets/reanalysis-era5-single-levels?tab=download. Field data sets are available online: Narrabeen Beach: http://narrabeen.wrl.unsw.edu.au/. Duck Beach: https://chldata.erdc.dren.mil/thredds/catalog/frf/geomorphology/elevationTransects/survey/data/catalog.html. Hasaki Beach: https://pari.mpat.go.jp/bdhome/Hasaki/. Torrey Pines Beach: https://datadryad.org/dataset/. doi:10.5061/dryad.n5qb383 Nha Trang and Grand Popo: https://zenodo.org/records/12805024

## Code availability

Code used to generate results will be made available upon request to the corresponding author.
