# Supplementary material for: Coastlines retreat tipping point under storm climate changes
Source: Sci Rep. 2026 Feb 24;16:10311. doi: 10.1038/s41598-026-40886-9 (PMC13031857; doi:10.1038/s41598-026-40886-9)
Supplement: Supplementary file 1 — Supplementary Information. [file 41598_2026_40886_MOESM1_ESM.pdf]

Coastlines retreat tipping point under storm climate changes

Marius Aparicio[1], Rafael Almar[2] , Laurent Lacaze[1] and Nicolas Robin[3]

[1] Univ Toulouse, Toulouse INP, CNRS, IMFT, Toulouse, France
[2] LEGOS (CNRS-IRD-CNES-University of Toulouse), Toulouse, France
[3]CEFREM Université de Perpignan Via Domitia 52 Avenue Paul Alduy Perpignan 66000, France

corresponding author: marius.aparicio@imft.fr

Storminess

Coastal storm climatology

Similar to the findings of Lobeto *et al.* (2024)[1] who used the 95th percentile of the wave distribution as the storm threshold, the estimated global storminess follows a similar trend: fewer but longer storms in the intertropical band, in contrast to the subtropical regions, which experience more sporadic storms throughout the year (Fig. SM1.A). The seasonal variability in storm activity aligns with the expected Austral vs. Boreal winter patterns, while the intertropical regions exhibit lower variability, reflecting the relatively stable storm-generation conditions characteristic of the Intertropical Convergence Zone[2].

Fig. SM.1.B-E illustrates the percentage of storm occurrences grouped by season. For both hemispheres, the majority of coastal storms occur during their respective winters Fig. SM.1.B, 1.D). Regions exposed to cyclonic tracks are also highlighted. For example, the Caribbean Sea and the Gulf of Mexico experience a significant number of storms during the cyclone season (June to October); as does the East Sea with the typhoon season (Fig. SM.1.E). Similarly, monsoonal activity is evident in the western Pacific, particularly around the Philippines and Indonesia (Fig. SM.1.B, 1.E).

The normalized interannual storminess variability (σ_{storm}) (Fig. SM.1.F) reveals a strong climate variability in the tropics, driven by atmosphere-ocean interactions and pronounced oceanic influences. It also highlights regions with significant interannual fluctuations, often linked to climate oscillations. Among these, the El Niño Southern Oscillation (ENSO) stands out. Due to its influence (among others) on sea surface temperatures, sea level, and surface gravity waves[3,4], ENSO influences the entire globe through multiple teleconnections and its impact on long-term sandy beach dynamics has been well-documented[5,6] . High STDs (0.65-0.7) in storm variability are evident along the Pacific coast of America and the Malay Archipelago, reflecting ENSO areas of influence. A strong correlation between the storm interannual variability and ENSO Index 3.4 oscillation was observed for the aforementioned locations ($0.3 < R^2 < 0.5$) (Fig. SM2). Fewer annual coastal storm events occur worldwide during positive ENSO phases (2 storms on average) compared to negative phases (4 storms on average). In the western part of the Indian Basin, the region influenced by the Indian Ocean Dipole (IOD)[7,8] is also marked. Finally, the most stable storm climates are observed in semi-enclosed seas and along east-facing coasts, such as those of western Canada, Mozambique and Uruguay.

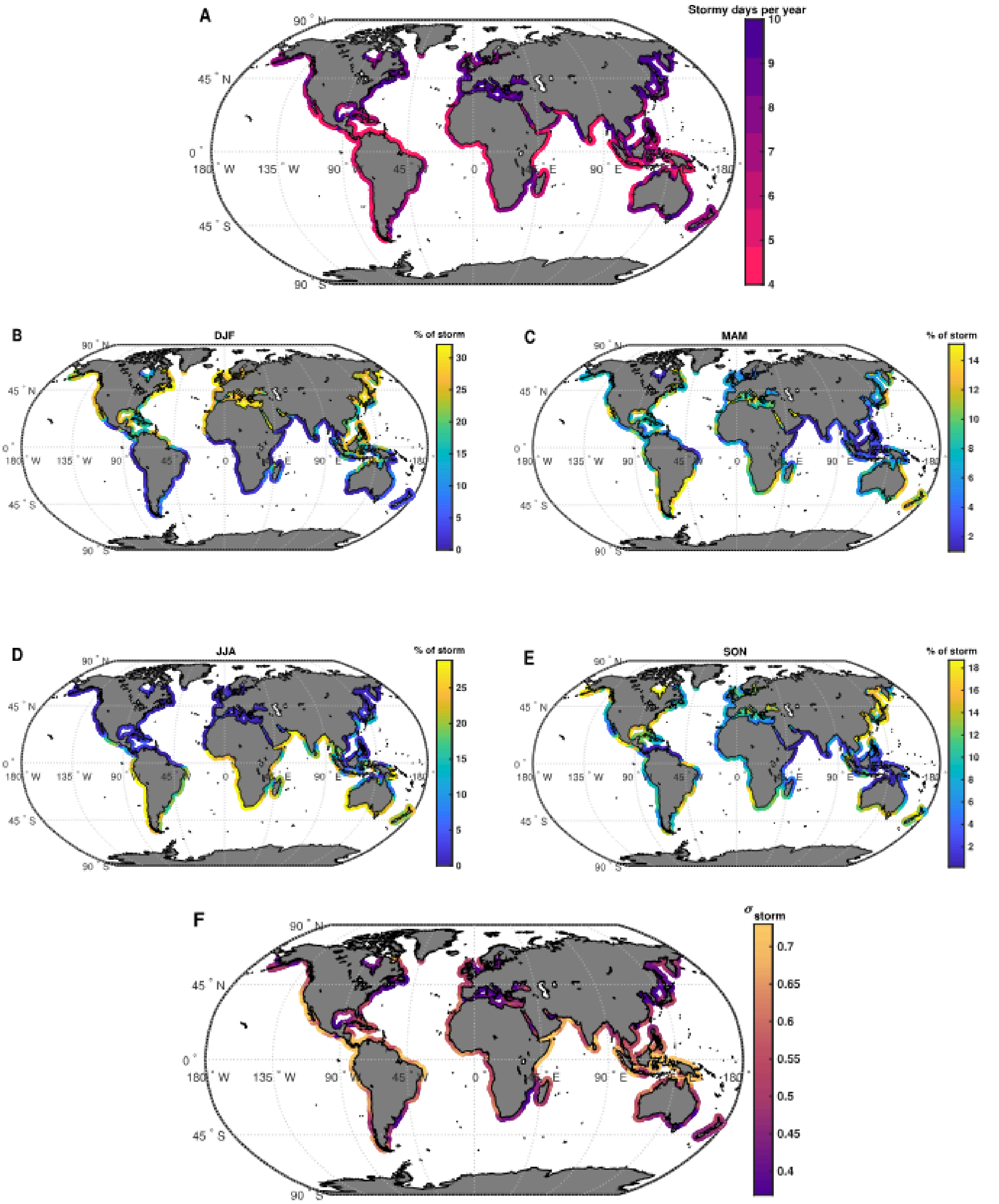

Figure SM1: *Global storminess and storm variability for seasonal and interannual scales.* *(A) Map presents the annual coastal storminess. (B-E) Maps illustrate the seasonal distribution of storm occurrences around the world, aggregated by percentage for each meteorological season: (B) DJF (December-January-February), (C) MAM (March-April-May), (D) JJA (June-July-August), and (E) SON (September-October-November). The distribution highlights the geographical variability in storm activity, with distinct patterns emerging in different regions depending on the season. (F) Map displays the interannual storminess variability across the globe normalized by the average number of storms over the period, estimated via the standard deviation (σ_{storm}) of storm activity. World maps were generated using MATLAB R2023b (https://matlab.mathworks.com).*

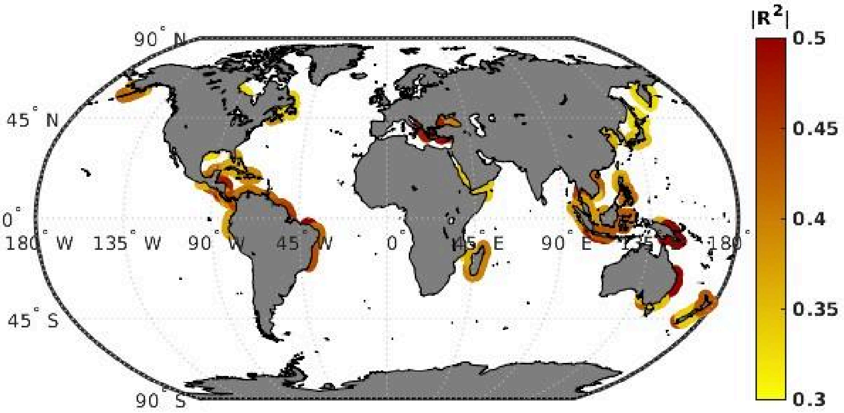

Figure SM2. |R^2| Between coastal storm interannual variability and ENSO phases.
The map displays the correlation coefficients between coastal storm variability and ENSO index 3.4 modulations, retaining only locations with a p-value < 0.05. The plot highlights a clear correlation between the interannual variability of coastal storms and ENSO, predominantly within the intertropical band. World maps were generated using MATLAB R2023b (https://matlab.mathworks.com).

Storm distribution, associated energy and duration

Fig. SM3 illustrates the latitudinal distribution of coastal storms detected during the 38-year monitoring period. The plot complements **Fig. SM1. A** by showing fewer storm occurrences within the intertropical region compared to the Northern Hemisphere.

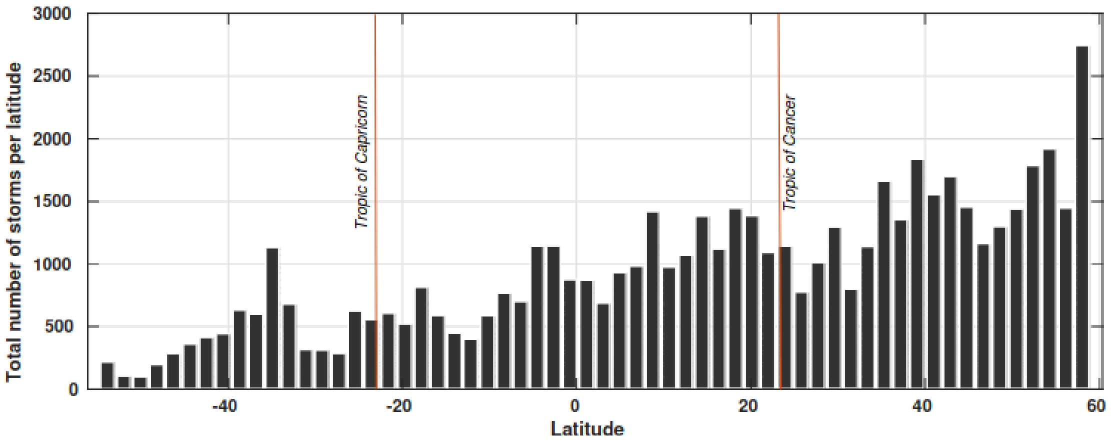

Figure SM3. ***Latitudinal Distribution of Storms Across the Globe.***
This bar chart presents the total number of storms per latitude, illustrating the distribution of storm occurrences from approximately 60°S to 60°N. The chart reveals a higher concentration of storms in mid-latitude regions, particularly around 40°N and 60°N, with fewer storms observed near the equator and at higher southern latitudes because of lesser continental proportion.

Fig. SM3 displays the storm wave energy distribution derived from the storm wave composites. As expected, latitudinal storm track bands exhibit higher storm wave energy than other regions, with the lowest storm wave energy observed in the intertropical band.

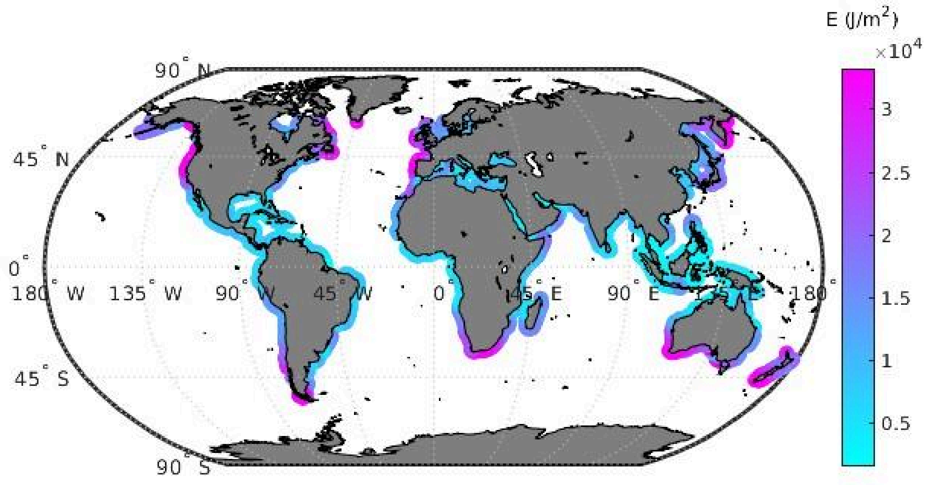

Figure SM4. Global Distribution of Storm Wave Energy.
This map illustrates the global distribution of storm wave energy along coastal regions. West facing coast located in the storm tracks have the highest typical storm wave energy. World maps were generated using MATLAB R2023b (https://matlab.mathworks.com).

Figure SM5.A presents the typical duration of storms lasting more than one day. It indicates that the longest-lasting storms, which can persist for up to three days, are concentrated around the equator. **Fig. SM5.B** shows the ratio (R) of 'one-day storms' to 'more-than-one-day storms,' providing an assessment of the prevalence of short- versus long-duration storm events across coastal regions. A ratio of $R > 1$ indicates dominance of short-duration storms, while $R < 1$ signifies regions mostly affected by long-lasting storms. The results reveal distinct differences between the intertropical band and extratropical regions, along with varying patterns between semi-enclosed seas, west-facing, and east-facing coasts.

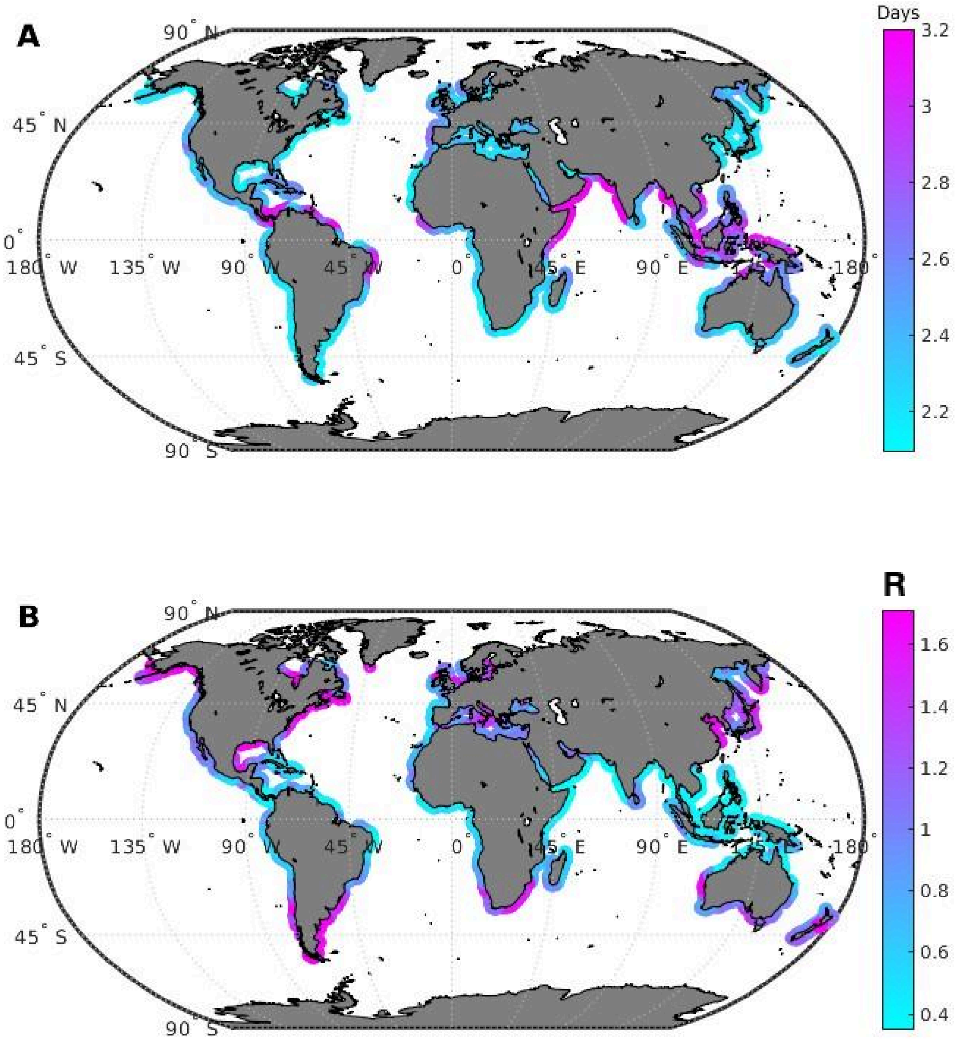

Figure SM5. Global Analysis of Storm Duration and Ratio of One-Day Storms to Multi-Day Storms.

(A) *The upper map shows the global distribution of the average duration of storm events that last for at least two days. This map highlights coastal regions more prone to extended storm events.*

(B) *The lower map illustrates the ratio of storm events lasting one day to those lasting at least two days or more. This map provides insights into the relative prevalence of short versus long-duration storm events across different coastal regions. World maps were generated using MATLAB R2023b (https://matlab.mathworks.com).*

Storm composite robustness through Confidence Index

A confidence index (CI) is defined to evaluate the robustness of the shoreline signal the storm composite, based on the number of storms retained after applying the selection criteria over the shoreline time series. This CI is used to assess the reliability of the composites (Fig. SM6) and to assign them a corresponding confidence weight.

$$CI = 100 * \frac{NSSi}{(<NSS> + \boldsymbol{\sigma})}$$

(1)

In (1), *NSS* represents the worldwide total number of shoreline signals retained to generate the storm composite after applying the selection criteria, *NSSi* refers to the number of shoreline signals retained for the storm composite at a specific location after applying the same criteria and $\boldsymbol{\sigma}$ is the standard deviation of *NMS.*

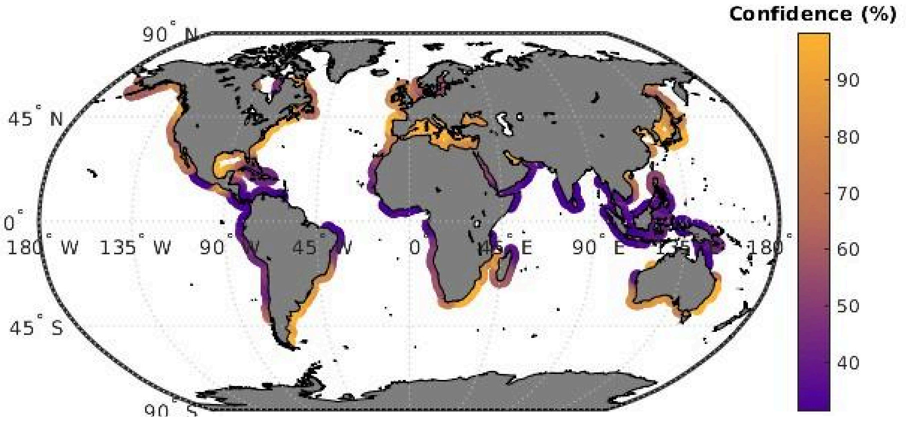

Figure SM6. Confidence Index for the robustness of the shoreline signal generated by the storm composites. *World maps were generated using MATLAB R2023b (https://matlab.mathworks.com).*

Error assessment of storm-induced erosion and post-storm recovery for the *in situ*-derived storm composites

Figures SM7–SM12 present the storm composites generated using the study's methodology across the four reference sites. Storms were categorized into four distinct groups:

1. **One-day storm**: A storm lasting only one day, without considering storm history.
2. **Several-day storm**: A storm persisting for at least two days, without considering storm history.
3. **Unique storm**: An isolated storm occurring outside a storm sequence. The time interval between two storms (**D**), above which a storm is considered isolated, corresponds to the estimated shoreline recovery time.
4. **Storm sequence**: A series of storms occurring within an interval shorter than **D**.

Storms are thus classified first by duration and subsequently by sequencing.

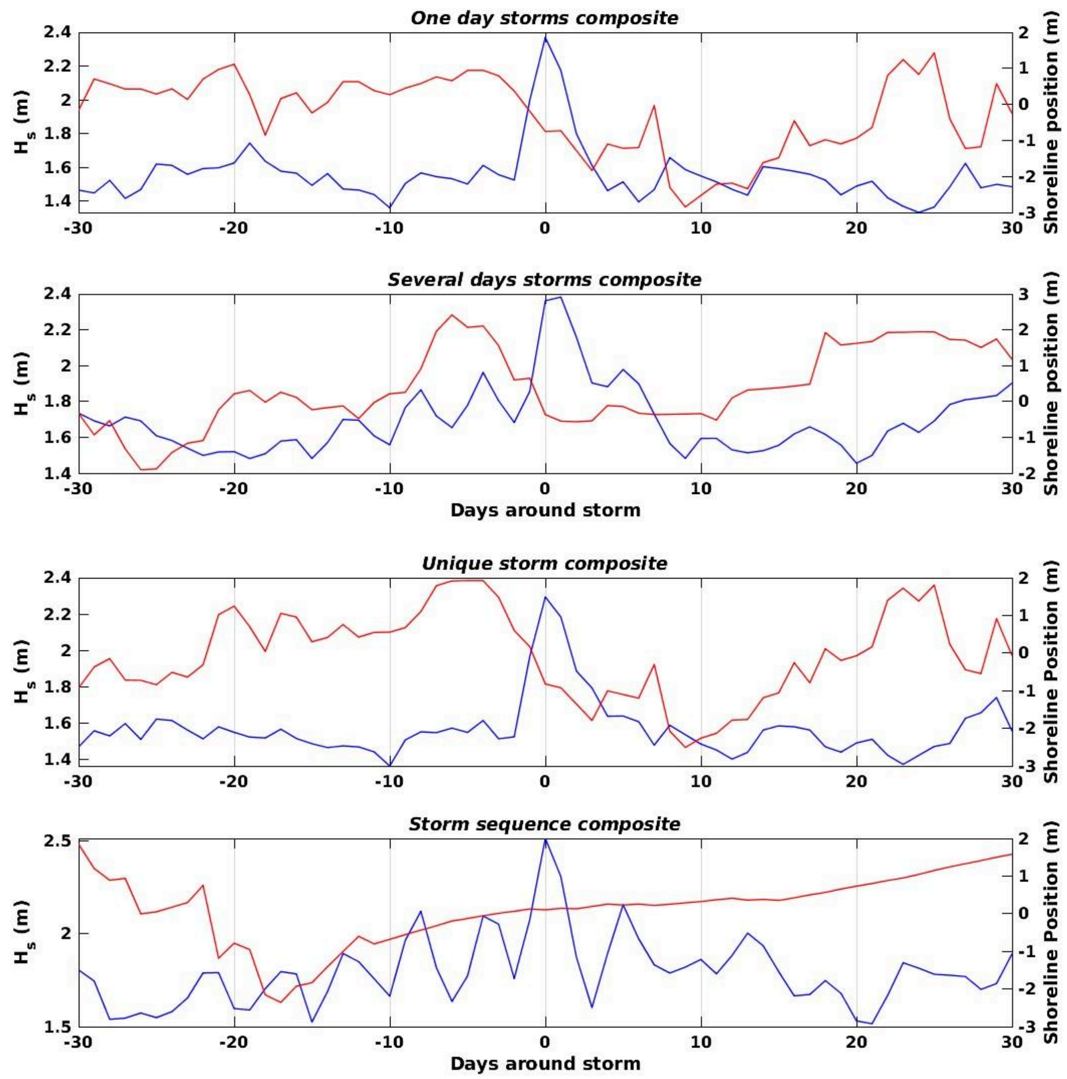

Figure SM7. Grand Popo beach storm composites

Storms are clustered by duration and sequencing in order to generate the wave height (blue) and shoreline position (red) composites. The storm sequence composite shows that the high wave sequence favors slow beach accretion at Grand Poppo.

Composites enable the statistical assessment of the typical shoreline response to different storm events. As shown in **Figures SM7–SM12**, storm wave height does not correlate with storm duration (i.e., long-lasting storms exhibit similar associated energy to short storms). The results also demonstrate that while each beach exhibits a distinct response to storms, all share a 'typical response' to coastal storms, characterized by a local recovery time and a range of storm-induced erosion. Moreover, both storm duration and storm sequencing influence shoreline retreat and recovery time. Notably, the impact of storm sequencing varies significantly among beaches. For example, some beaches, like Nha Trang (**Figure SM8**), exhibit greater resilience, with nearly complete recovery between successive storms within a sequence.

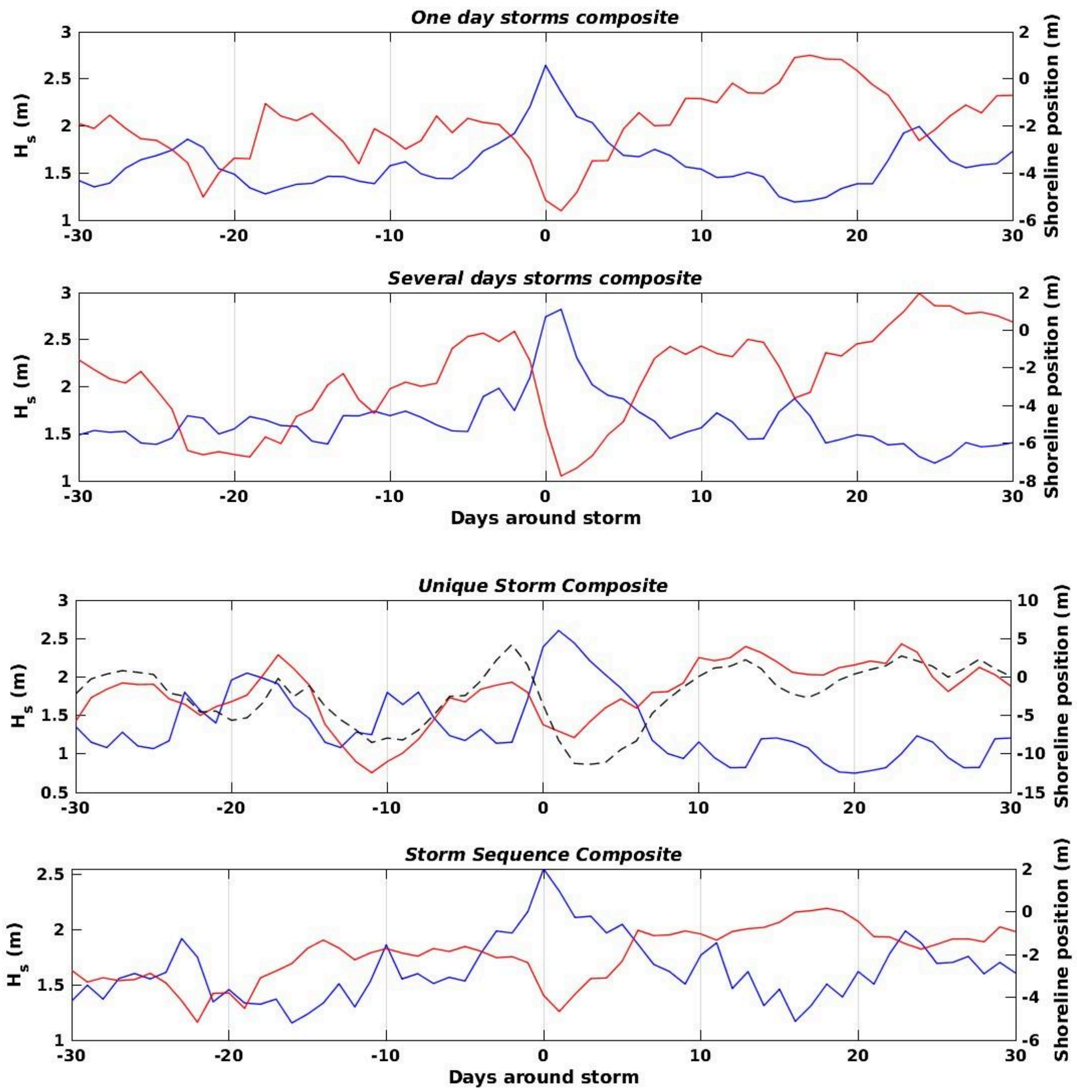

Figure SM8. Nha Trang beach Storm Composites

Storms are clustered by duration and sequencing in order to generate the wave height (blue) and shoreline position (red) composites. For the unique storm composite, Shoreline position is shown with (black-dashed) and without (red) an extreme unique storm impact due to severe cyclonic impact[9]*.*

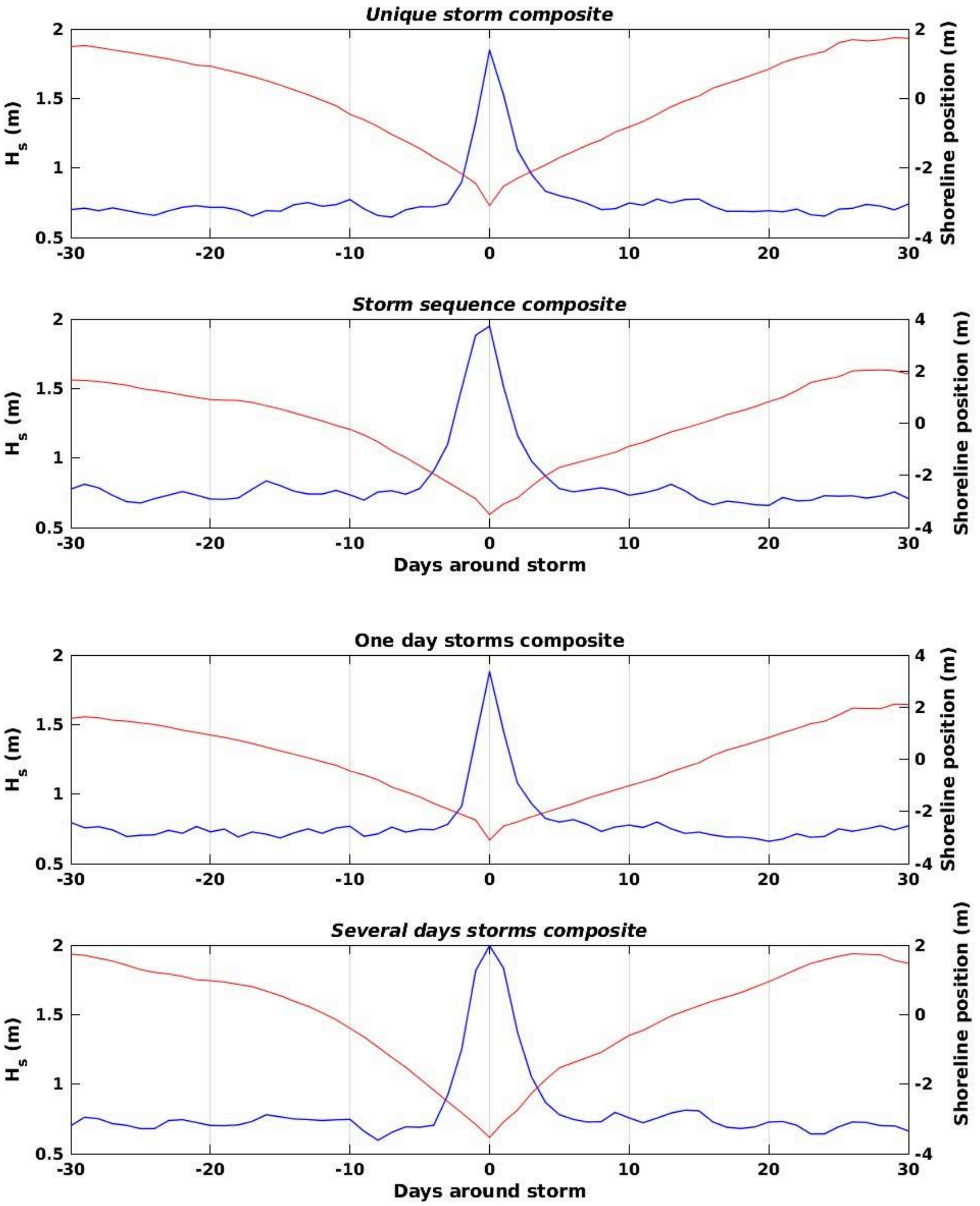

Figure SM9. Narrabeen beach storm composites
Storms are clustered by duration and sequencing in order to generate the wave height (blue) and shoreline position (red) composites.

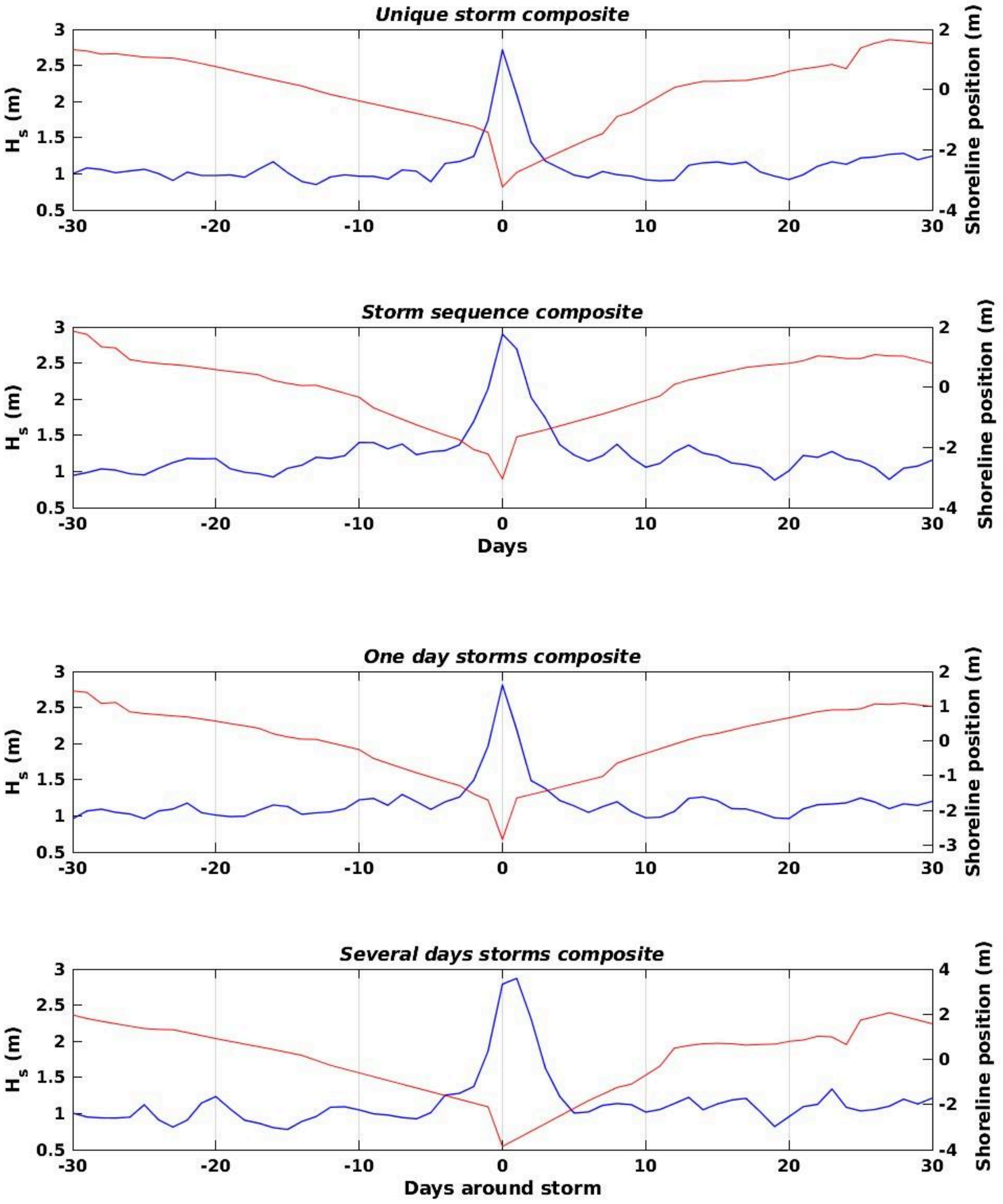

Figure SM10. Duck beach storm composites

Storms are clustered by duration and sequencing in order to generate the wave height (blue) and shoreline position (red) composites.

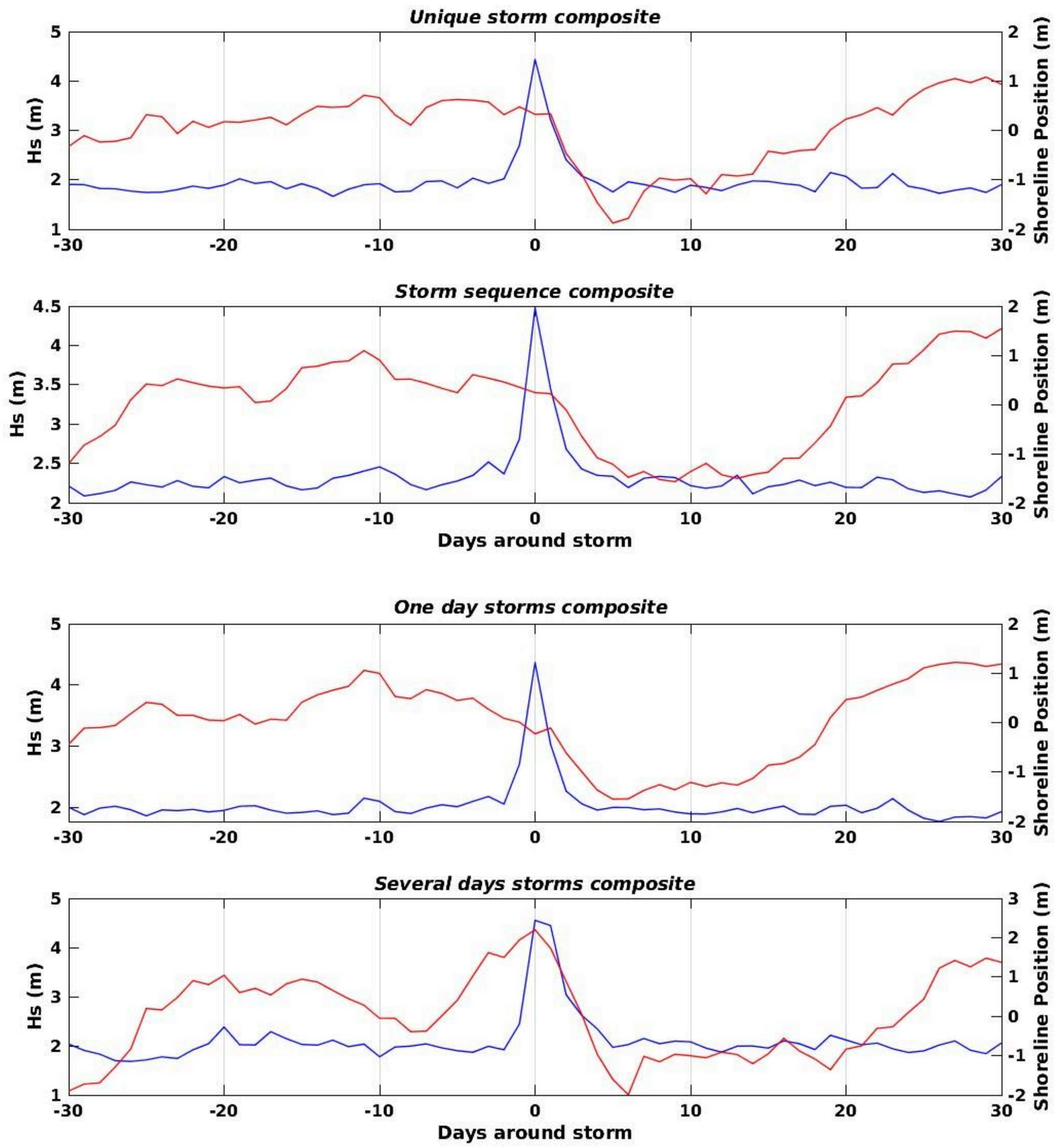

Figure SM11. Hasaki beach storm composites

Storms are clustered by duration and sequencing in order to generate the wave height (blue) and shoreline position (red) composites.

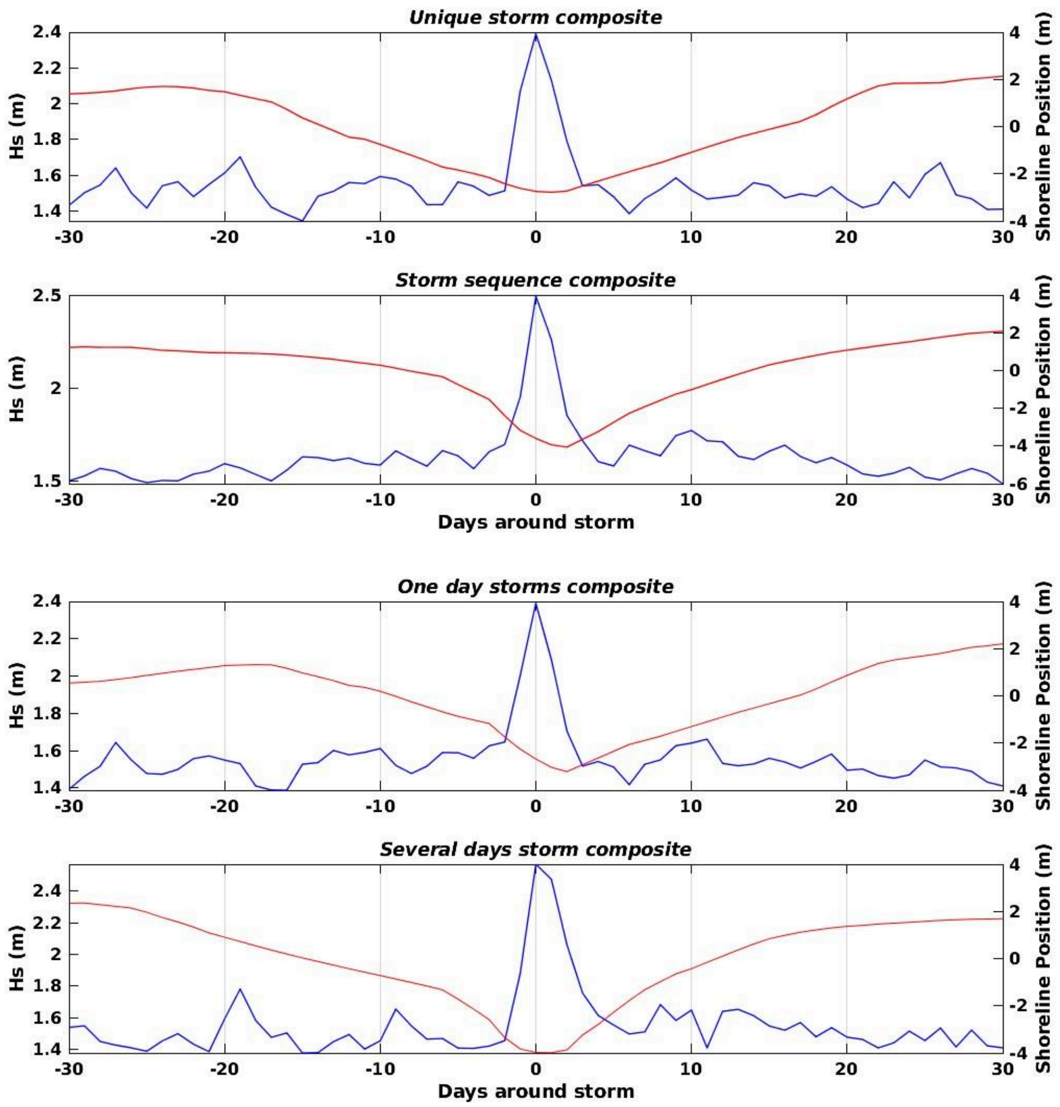

Figure SM12. Torrey Pines storm composites
Storms are clustered by duration and sequencing in order to generate the wave height (blue) and shoreline position (red) composites.

Offshore significant wave height statistical analysis

Skewness (S) provides critical information about the time spent in storm wave conditions (Fig. SM13.A). The sign of S indicates the distribution's asymmetry: $S > 0$ denotes a bulk of data concentrated on the left with a tail to the right, while $S < 0$ indicates the opposite. When $S = 0$, the distribution is unimodal (not necessarily symmetric) suggesting a relatively uniform wave climate with low occurrence of extreme events.

As expected, (Fig. 2.A) shows skewness values ranging from near-zero to positive S, as no coastal area on Earth experiences constant extreme wave heights (i.e. $S < 0$).
The intertropical band is distinctly differentiated from the extratropics, and the global skewness map correlates strongly with annual storm frequency (Fig. SM1.A). Locations with low skewness ($0 < S < 1$) coincide with regions experiencing the lowest storm frequency per year. On the other hand, areas with the highest storm rate per year exhibit the most skewed distribution ($S > 1$). Interestingly, the west-facing coasts of the Northern Hemisphere,

positioned within major storm tracks, do not have the most skewed wave distributions. These areas, frequently exposed to large atmospheric storms and persistently high wave energy, exhibit reduced skewness due to the relative uniformity of wave heights. To illustrate the wave distributions and their associated skewness, three representative global locations are plotted in Fig. SM13.C.

Kurtosis (K), a scaled version of the fourth moment of the distribution, provides insights into the spread and thickness of its tails, which reflect the presence of outliers. If K < 0, outliers are rare, and the distribution's values are tightly clustered around the mean. When K = 0, the tails resemble those of a typical normal distribution. Conversely, K > 0 indicates that outliers are relatively distant from the mean, with the magnitude of K reflecting the extent of this deviation. Applied to the offshore significant wave height distribution (Fig. SM13.B), the kurtosis analysis reveals patterns consistent with the skewness results. Locations with high frequency storms and strongly skewed wave heights distributions also exhibit the highest kurtosis values. This indicates that significant wave heights and the associated wave energy in these regions deviate substantially from the statistical wave equilibrium conditions.

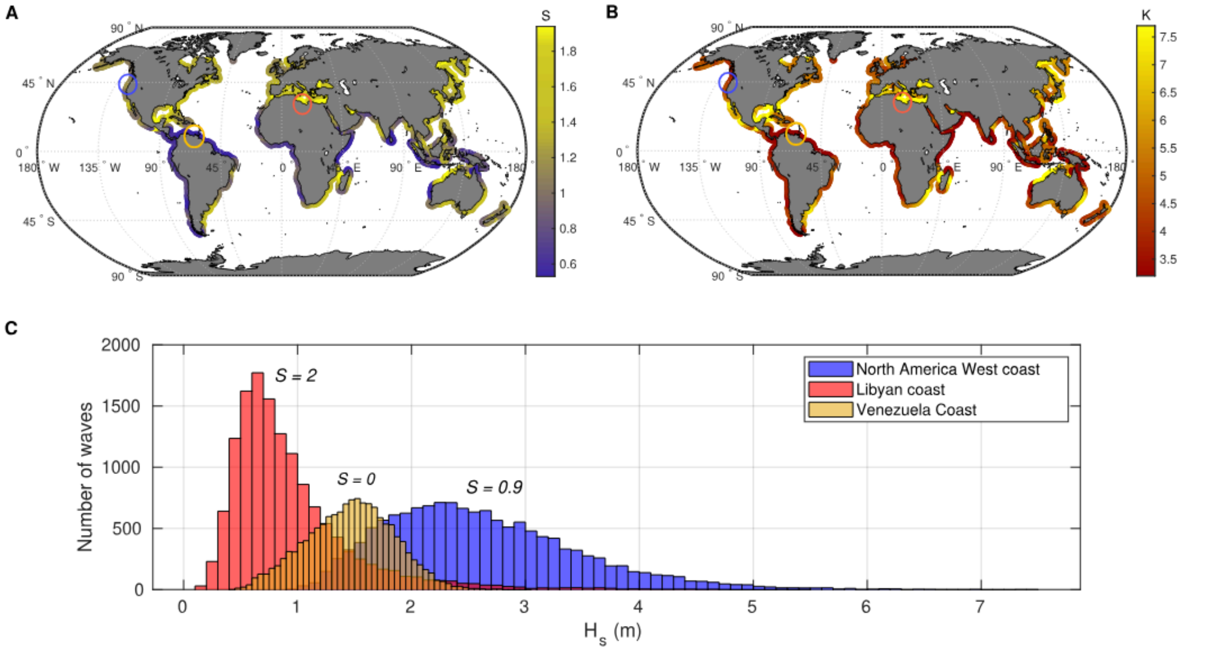

Figure SM. 13. Global wave skewness and kurtosis with corresponding histograms. *This figure illustrates the global distribution of wave skewness (S) and kurtosis (K) and provides examples of wave height histograms for selected coastal regions. The top maps show the global distribution of wave skewness (A) and kurtosis (B). The bottom histogram displays wave height occurrence for three different coastal regions: the west coast of North America (blue), the coast of Libya (orange), and the coast of Venezuela (yellow). The skewness (S) values are provided for each region, illustrating how the distribution shape changes with increasing skewness: the higher the skewness, the more the distribution mass shifts to the left, with a longer tail on the right. World maps were generated using MATLAB R2023b (https://matlab.mathworks.com).*

Non-sandy coast filtering at global scale

Accurately identifying sandy shorelines while excluding remnants of other coastline types remains a significant challenge when working with large satellite-derived datasets. To address this, the monthly satellite-derived shoreline dataset is pre-processed to exclude pebbly and gravel beaches.

Given that their dynamics are strongly influenced by tides, substantial sediment deposition, and water density plumes, a database of muddy coastlines compiled by Hulskamp *et al.*, (2023)[10] is utilized to identify and remove salt marshes and mangrove swamps, which collectively account for nearly 15% of the world's coastline.

Lastly, a density filter is applied to the remaining sandy coastlines to eliminate lagoons, estuaries, and highly embayed environments sheltered from wave action. This filter assesses coastline variability by calculating the mean variability of any three consecutive points; if the value is below a specified threshold, the sites are excluded from the dataset.

$$\overline{\sigma} = \frac{\sigma_{lat} + \sigma_{lon}}{2} \leq T \qquad (2)$$

Where $\overline{\sigma}$ represents the average standard deviation (mean variability) of latitudinal and longitudinal coordinates, σ_{lat} , σ_{lon} are the standard deviations of the latitudes and longitudes within the range (3 consecutive points) and T is the threshold, set to 0.32 after various sensitivity tests. To illustrate the overall filtering, some regional locations before and after the density filter are shown in **Fig. SM14**.

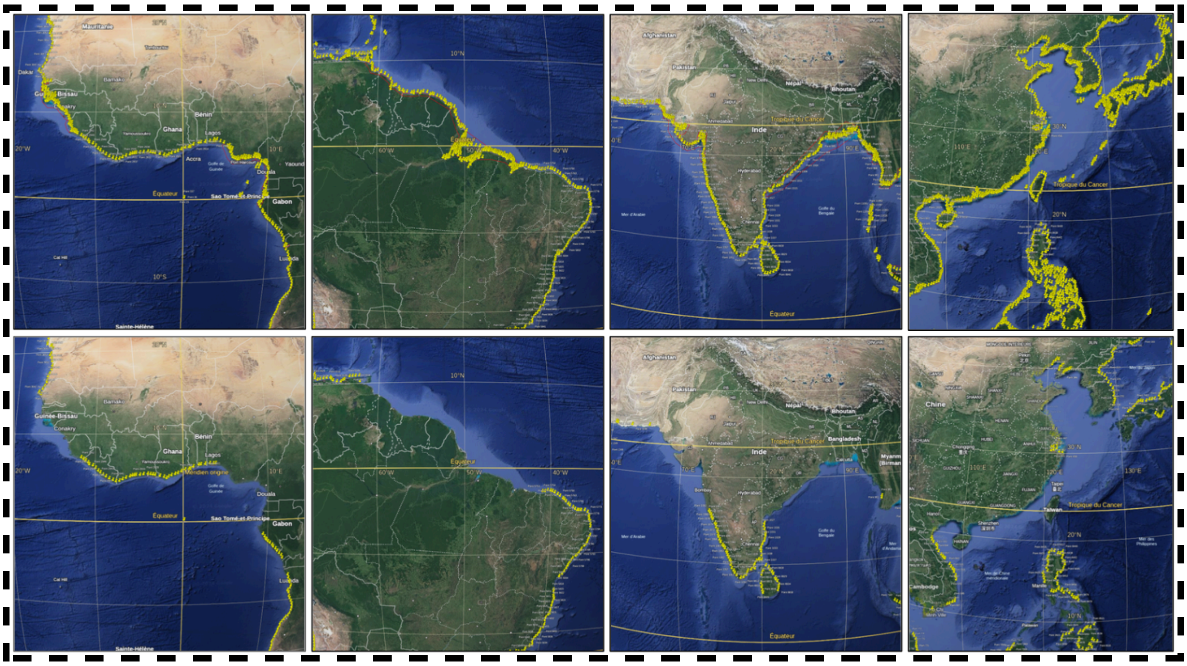

Figure SM14. Regional Comparison of Global Sandy Coastline Locations Before and After Spatial Filtering of Muddy Shores, Lagoons, **Highly Embayed Environments** ***and Estuaries.***

Upper panels show the initial dataset of identified sandy coastline locations (pebble and

gravel beaches are already filtered out). Lower panels show the post-filtered dataset, where muddy shores and estuaries have been filtered out. Imagery © 2023 CNES/Airbus, Map data © 2023 Google.

Shoreline recovery rate and post-storm wave energy

The collection of shoreline recovery speed after storm-induced erosion at a wide range of locations worldwide revealed a power law relationship between the latter and post-storm wave energy (revealed being similar to the mean wave energy on site) namely E_w (J.m.$^{-2}$). The scatter-plot is shown in **Fig. SM15.**

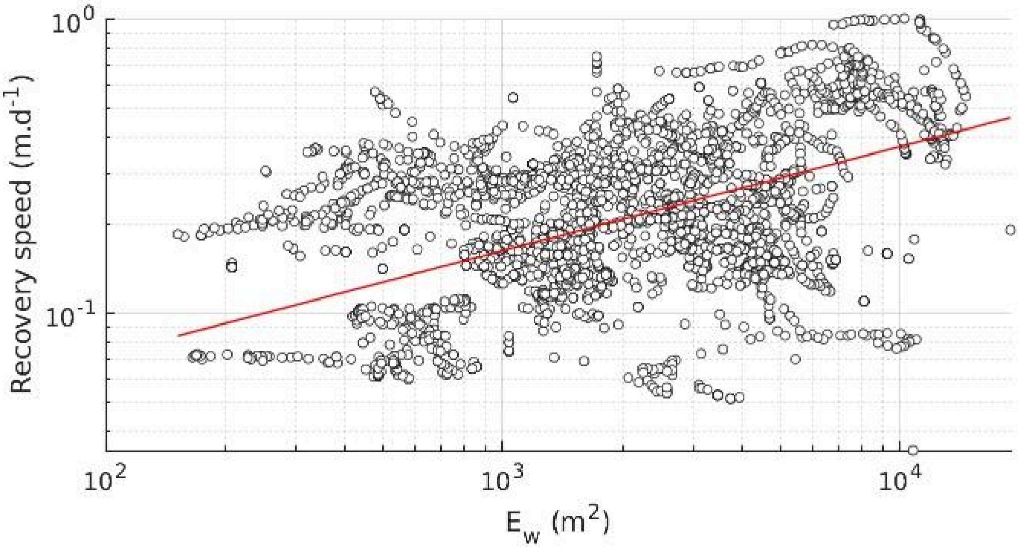

Figure SM15. Log-log scatter plot of the coastal recovery speed as a function of the mean wave energy.

Figure shows the relationship between mean wave energy and the post-storm shoreline recovery speed, suggesting that coasts subjected to higher mean wave energies tend to recover slightly faster. However, as indicated by the data spreading, there is substantial variability that may be due to other environmental factors blurring the correlation.

Each point represents a different coastal location. The red line indicates the overall trend used to derive a and k (see Eq. 2 in main manuscript).

Distribution of the data used to estimate trends

Fig. SM16 shows that the data distribution presents relatively low skewness and kurtosis with very few outliers identified by the Interquartile Range (IQR) method (red cross), which have been ignored to compute the trends. This confirms that the data distribution is adequate to apply the IQR-method.

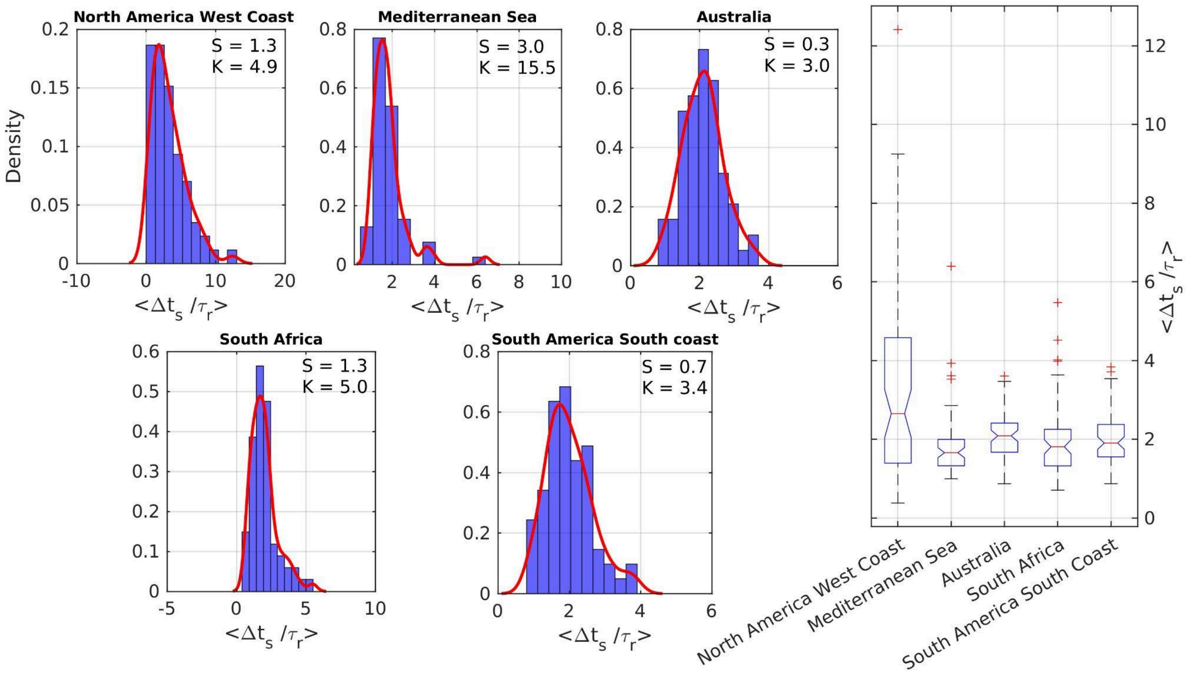

Figure SM16. Histogram of the yearly averaged tipping point ($\Delta t_s/\tau_r$) values along with the boxplot for each hotspots flagged in Fig. 3 of the main manuscript.

Histogram subpanels show that the data distribution presents relatively low skewness and kurtosis. Box-plot subpanels show the very few outliers identified by the IQR-methods (red cross), which have been ignored to compute the trends.

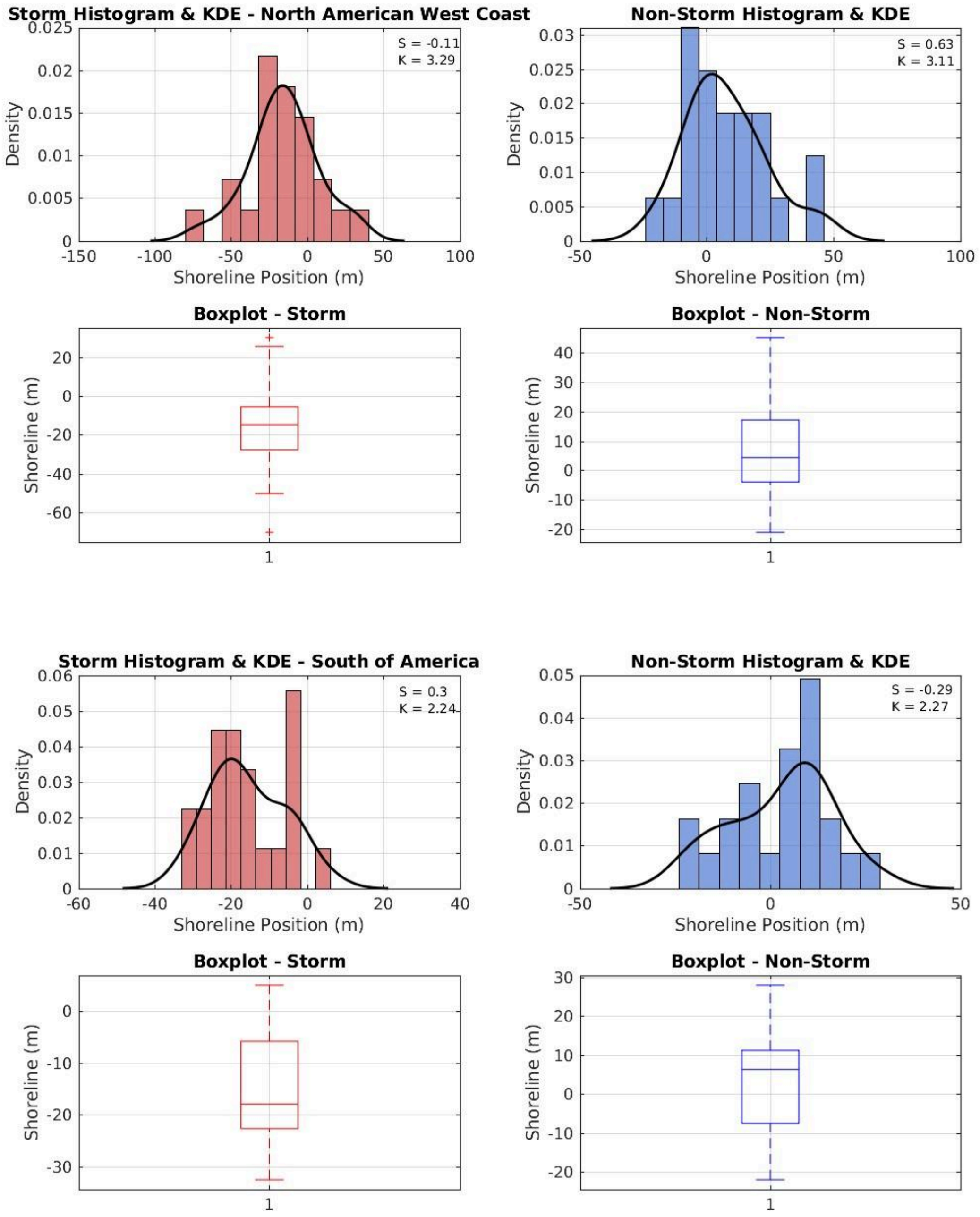

Figure SM17. Histogram of the yearly averaged shoreline position for storm and non-storm season along with the boxplot for each of them at the two locations shown in Fig. 4 of the main manuscript

Linear regression between CSS and all its component

Fig. SM 18 presents the regression coefficients and the correlation matrix used to derive the interpretation of the evolution of the CSS through time via the coevolution of Hs_{storm}, Hs_{mean} and NS the number of storms (here named storm count).

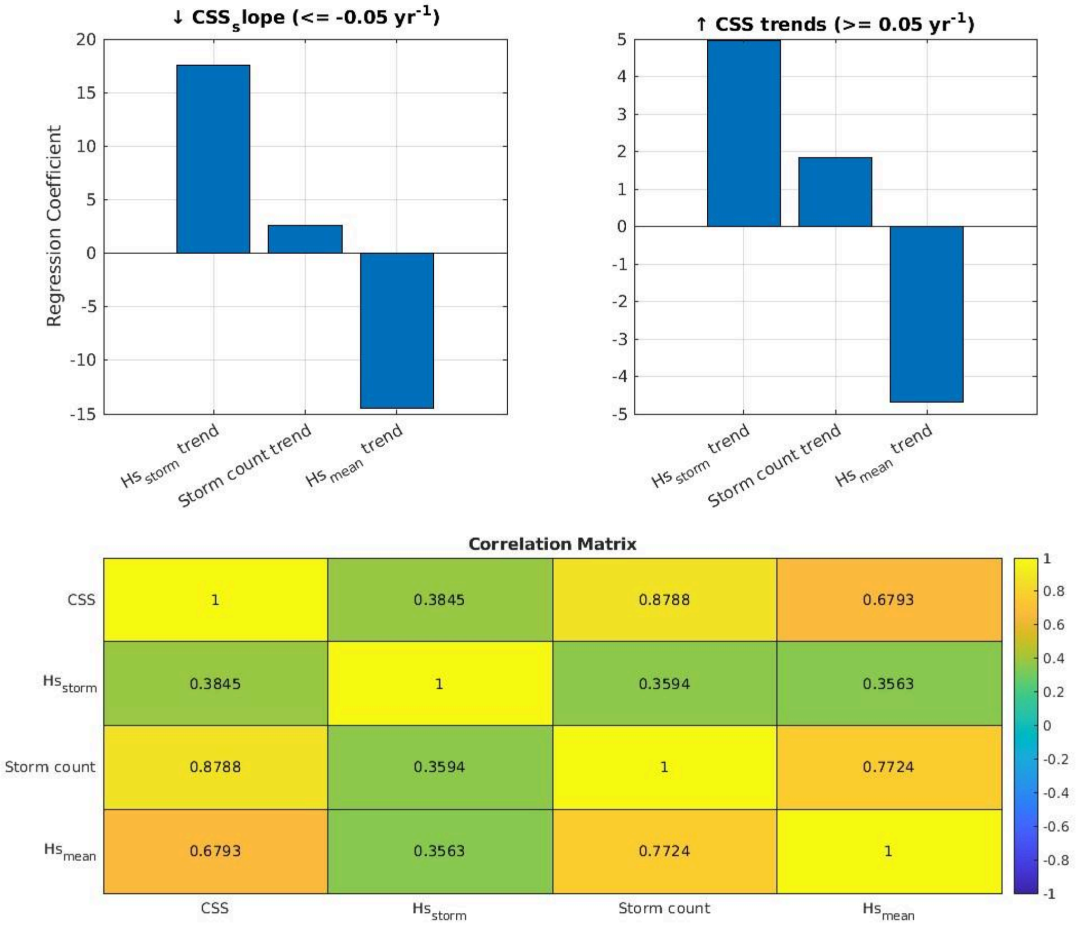

Figure SM18. Regression coefficient and correlation matrix for each component of the CSS.

Seasonal climatology of the storm tipping point

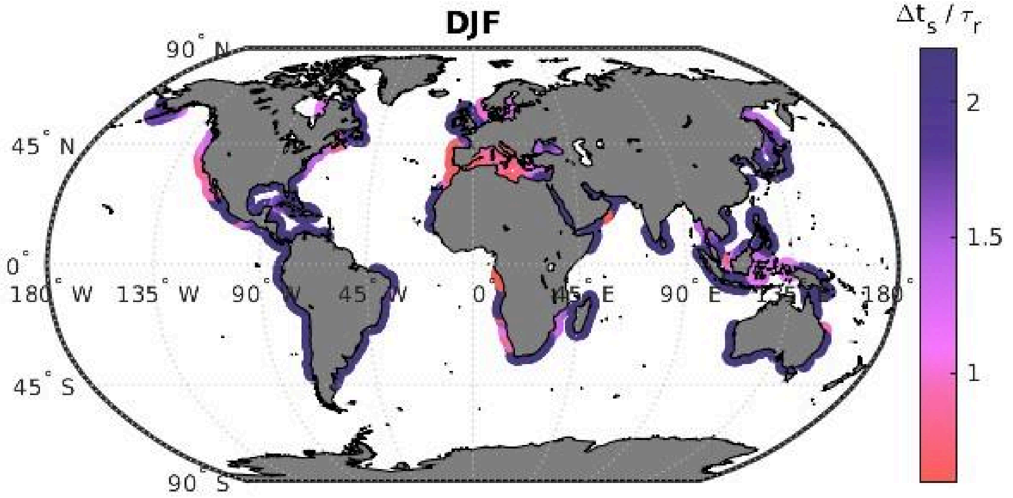

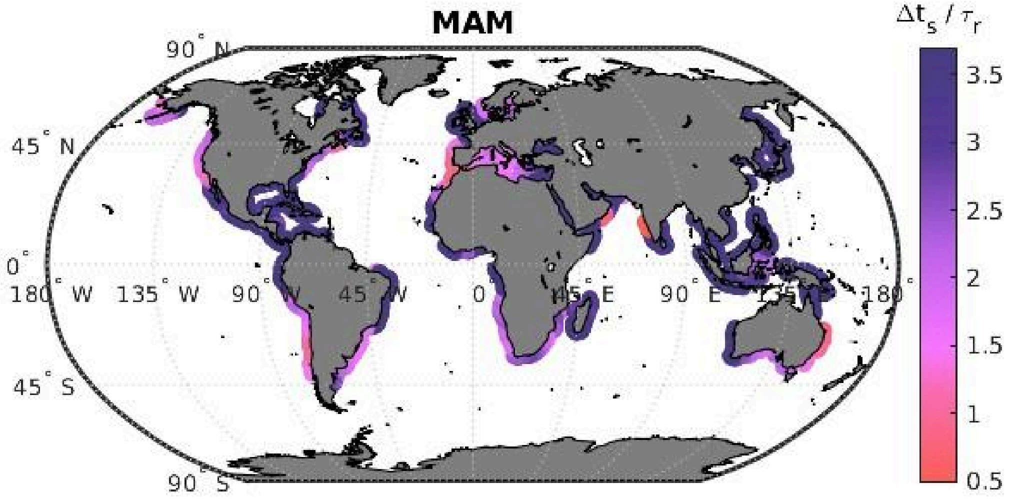

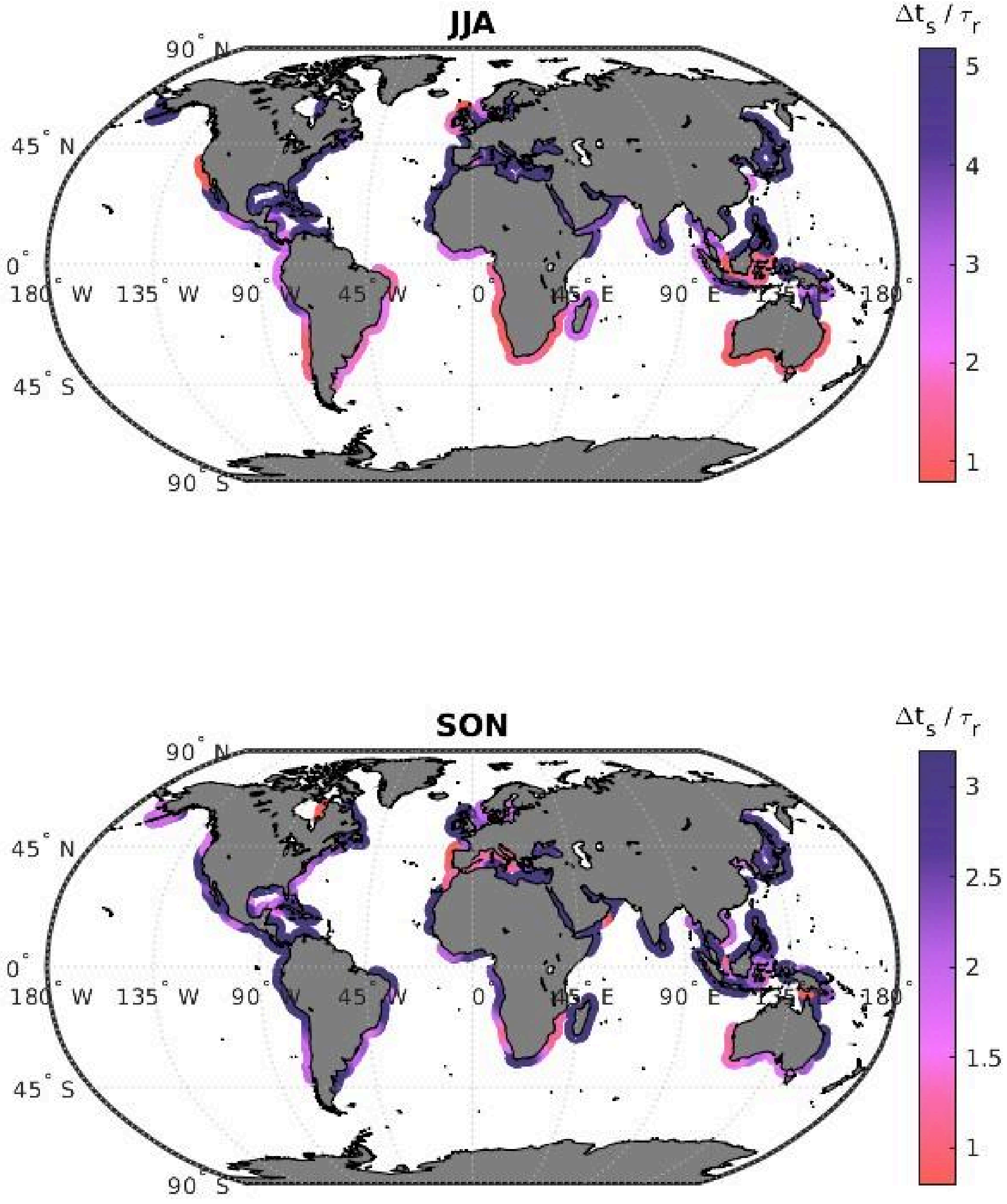

Figure SM19. Seasonal climatology of the tipping point.

Maps reveal that the tipping point is mostly crossed during the storm season of each location (Fig. SM1.B-E). World maps were generated using MATLAB R2023b (https://matlab.mathworks.com).

SDS dataset validation along North West American Coast

Text from Almar et al (in review), provided here to accompany Fig. SM20:

To ensure a direct comparison with the global waterline dataset used in this study, the high-resolution (275 m-spaced), tide-corrected shoreline dataset (HRTC), from Graffin et al. (2025), dataset was spatially averaged to match the coarser resolution GlobC dataset and processed using the same temporal filtering techniques to isolate seasonal and interannual components. A total of 225 co-located transects-matched within a ±0.025° latitude tolerance—were analyzed along the Pacific coastline. The resulting time series show strong agreement between the two datasets, with an average Pearson correlation coefficient of 0.82 and a mean RMSE of 2.4m across the full latitudinal range (**Fig SM20.d**), supporting the robustness of the large-scale signals captured by the GlobC dataset. In particular, the phase and amplitude of the seasonal signal are well represented, and the deseasonalized time series indicates that the interannual variability (**Fig SM20.e**) is also correctly captured. This global dataset was designed to analyze large-scale regional to global patterns and should be applied within that intended framework. The 0.27° resolution of the global transects may appear relatively coarse. However, with access to a high-resolution dataset, we were able to assess the sensitivity of regional aggregates (computed over 3° windows) to changes in alongshore resolution—ranging from the finest scale of 250m to the coarsest of 0.5° (approximately 50km). As shown in **Fig SM20.f**, the results indicate limited sensitivity to resolution changes, with a correlation loss of only 10%—from 1.0 to 0.9—relative to the finest-scale time series.

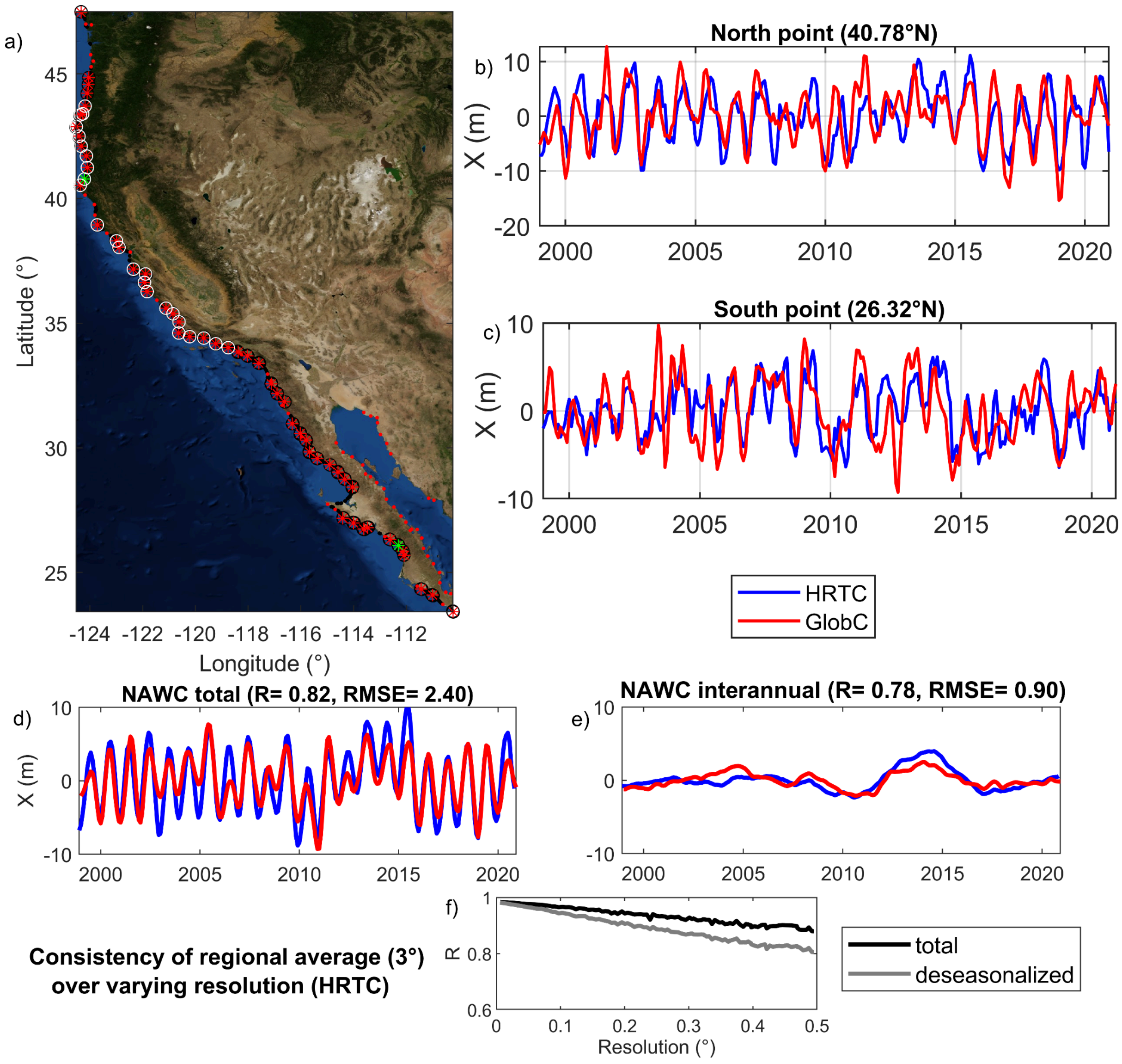

Figure SM20: ***Figure from Almar et al. (in review).*** ***Comparison of our global coarse (GlobC) dataset with the tide-corrected shoreline dataset (HRTC).*** *This dataset covers the western coast of North America (NAWC) - see a). black and red dot stands for HRTC and GlobC points respectively, matching locations are shown as stars. North and South locations taken for comparison in b) and c) appear as green stars. White circles show the locations taken for the regional average shown in d) total signal, and e) interannual signal. f) shows the result of correlating regional (3°) average with full resolution of HRTC with same average with reduced resolution (GlobC uses 0.27° median resolution).*

References

[1]. Lobeto, H. *et al.* (2024) « Global coastal wave storminess », *Scientific Reports*, 14(1), p. 3726. Disponible sur: https://doi.org/10.1038/s41598-024-51420-0.

[2]. Holton, J.R. et Hakim, G.J. (2013) *An Introduction to Dynamic Meteorology*. Academic Press.

[3]. Gouirand, I. et Moron, V. (2003) « Variability of the impact of El Niño–southern oscillation on sea‐level pressure anomalies over the North Atlantic in January to March (1874–1996) », *International Journal of Climatology*, 23(13), p. 1549‑1566. Disponible sur: https://doi.org/10.1002/joc.963.

[4]. Odériz, I. *et al.* (2020) « El Niño‐Southern Oscillation Impacts on Global Wave Climate and Potential Coastal Hazards », *Journal of Geophysical Research: Oceans*, 125(12), p. e2020JC016464. Disponible sur: https://doi.org/10.1029/2020JC016464.

[5]. Mortlock, T.R. et Goodwin, I.D. (2016) « Impacts of enhanced central Pacific ENSO on wave climate and headland-bay beach morphology », *Continental Shelf Research*, 120, p. 14‑25. Disponible sur: https://doi.org/10.1016/j.csr.2016.03.007.

[6]. Almar, R. *et al.* (2023) « Influence of El Niño on the variability of global shoreline position », *Nature Communications*, 14(1), p. 3133. Disponible sur: https://doi.org/10.1038/s41467-023-38742-9.

[7]. Saji, N. et Yamagata, T. (2003) « Possible impacts of Indian Ocean Dipole mode events on global climate », *Climate Research*, 25, p. 151‑169. Disponible sur: https://doi.org/10.3354/cr025151.

[8]. Saji, N.H. *et al.* (1999) « A dipole mode in the tropical Indian Ocean », *Nature*, 401(6751), p. 360‑363. Disponible sur: https://doi.org/10.1038/43854.

[9]. Thuan, D.H. *et al.* (2016) « Typhoon Impact and Recovery from Continuous Video Monitoring: a Case Study from Nha Trang Beach, Vietnam », *Journal of Coastal Research*, 75(sp1), p. 263‑267. Disponible sur: https://doi.org/10.2112/SI75-053.1.

[10]. Hulskamp, R. *et al.* (2023) « Global distribution and dynamics of muddy coasts », *Nature Communications*, 14(1), p. 8259. Disponible sur: https://doi.org/10.1038/s41467-023-43819-6.
